# Engineering Neutrophil Vesicles for Synergistic Protection against Ischemia/Reperfusion Injury after Lung Transplant

**DOI:** 10.1002/advs.202506127

**Published:** 2025-08-14

**Authors:** Hao‐Xiang Yuan, Yu‐Yun Ye, Pu Shen, Jie Zhang, Qian‐Fang Meng, Ying Chen, Xin Xu, Xuan‐Lin Zhang, Lang Rao, Zhi‐Jin Fan, Jian‐Xing He

**Affiliations:** ^1^ Department of Thoracic Surgery and Oncology the First Affiliated Hospital of Guangzhou Medical University State Key Laboratory of Respiratory Disease & National Clinical Research Center for Respiratory Disease Guangzhou 510120 China; ^2^ Institute for Engineering Medicine Kunming Medical University Kunming 650500 China; ^3^ Department of Anesthesiology The First Affiliated Hospital Sun Yat‐sen University Guangzhou 510080 China; ^4^ Institute of Chemical Biology Shenzhen Bay Laboratory Shenzhen 518132 China; ^5^ Southern Medical University Guangzhou 510120 China

**Keywords:** cellular vesicles, ferroptosis, lung transplantation, neutrophil, ROS‐responsive

## Abstract

Lung transplantation (LTx) is a life‐saving procedure for patients with end‐stage respiratory failure; however, primary graft dysfunction (PGD), primarily induced by ischemia/reperfusion injury (IRI), remains a major complication. Although ex vivo lung perfusion (EVLP) improves preservation, clinical translation remains challenging owing to IRI complexity. Here, a novel approach is presented to mitigate lung IRI by developing of neutrophil‐derived ROS‐responsive cellular vesicles (SOD2‐Fer‐1@CVs). This hybrid system integrates superoxide dismutase 2 (SOD2)‐overexpressing neutrophil nanovesicles with ROS‐responsive liposomes loaded with ferrostatin‐1 (Fer‐1), a potent ferroptosis inhibitor. SOD2‐Fer‐1@CVs enabled targeted delivery to inflamed tissues and high oxidative stress environments, enabling ROS‐triggered release of SOD2 and Fer‐1. The SOD2‐Fer‐1@CVs system mechanistically targeted the core pathological pathways of IRI, including oxidative stress alleviation, adsorption and neutralization of pro‐inflammatory cytokines, ferroptosis suppression, and restoration of endothelial barrier integrity, with concurrent promotion of macrophage M2 polarization. Using the proprietary small‐animal EVLP platform, the therapeutic administration of SOD2‐Fer‐1@CVs significantly mitigated of reperfusion‐related pathologies and improved graft performance, including enhanced oxygenation, reduced airway resistance, and restored lung compliance, attenuating lung injury after LTx. This study established a novel nanotherapeutic strategy that synergizes with EVLP to address multifactorial IRI, showing high translational potential for improving donor lung quality and LTx outcomes.

## Graphical abstract

Engineered neutrophil‐derived vesicles (SOD2‐Fer‐1@CVs) co‐delivering antioxidant and ferroptosis‐inhibitory agents enable inflammation‐targeted, ROS‐responsive therapy for ischemia–reperfusion injury in lung transplantation. Synergizing with ex vivo lung perfusion, this strategy alleviates oxidative stress and inflammation, restores vascular integrity, and improves graft function, offering translational potential for enhancing lung transplant outcomes.

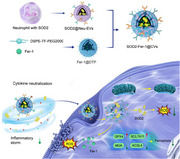

## 1 Introduction

Lung transplantation (LTx) improves survival and quality of life in end‐stage respiratory failure; however, its application is challenged by primary graft dysfunction (PGD), affecting more than 30% of the recipients and elevating mortality.^[^ ^1^, ^2^ ^]^ PGD manifests as acute lung injury with hypoxemia and diffuse infiltrates,^[^ ^3^, ^4^, ^5^ ^]^ driven primarily by ischemia‐reperfusion injury (IRI). IRI initiates alloimmune responses and is a key risk factor for chronic graft dysfunction. Although mechanistic insights into lung IRI have advanced,^[^ ^6^, ^7^, ^8^ ^]^ clinical application is hindered by its complexity. Multidimensional approaches targeting the diverse pathological processes of IRI hold promise for the prevention and treatment of PGD, offering potential improvements in LTx outcomes.

IRI drives interconnected pathological cascades.^[^ ^3^, ^4^, ^9^, ^10^ ^]^ Endothelial mechanosensing of ischemia triggers reactive oxygen species (ROS) overproduction and oxidative stress,^[^ ^11^, ^12^ ^]^ which induce DNA damage and damage‐associated molecular patterns, amplifying programmed cell death (e.g., ferroptosis).^[^ ^13^, ^14^ ^]^ Macrophages (M1 pro‐inflammatory and M2 anti‐inflammatory phenotypes) play a pivotal role in PGD pathogenesis.^[^ ^15^ ^]^ Post‐transplant endothelial injury activates innate immunity, promoting M1 polarization that results in the recruitment of donor/recipient monocytes^[^ ^16^ ^]^ and release of cytokines (e.g., IL‐6, TNF‐α) alongside neutrophil‐activating chemokines. This self‐perpetuating cycle of oxidative stress, inflammation, and cell death establishes IRI as a critical driver of PGD.^[^ ^3^, ^4^, ^5^ ^]^ Therapeutic strategies targeting these core mechanisms may disrupt IRI progression and improve graft survival.

Ex vivo lung perfusion (EVLP) has emerged as a key strategy to reduce PGD during lung transplantation.^[^ ^17^, ^18^, ^19^, ^20^ ^]^ This technique maintains donor lungs with oxygen/nutrient‐rich perfusion, thereby enabling functional assessment and tissue repair.^[^ ^18^ ^]^ However, EVLP also activates IRI‐like inflammatory/cell death pathways (cell death, mitochondrial dysfunction, and oxidative stress).^[^ ^13^, ^14^, ^21^ ^]^ EVLP‐compatible interventions, such as cytokine adsorption, adenosine‐enhanced metabolism, complement inhibition, and stem cell therapy, may alleviate lung injury. Despite therapeutic potential,^[^ ^18^, ^22^ ^]^ their clinical application is constrained by the focus on a single pathway. Current therapies (e.g., dexamethasone) have limitations, including low lung bioavailability and transient antioxidant effects.^[^ ^23^ ^]^ The development of multi‐mechanism interventions targeting concurrent IRI pathways is critical for enhancing graft protection during LTx.

Nanoscale‐targeted drug delivery has demonstrated immense potential for mitigating lung tissue injury and promoting functional recovery.^[^ ^24^, ^25^, ^26^, ^27^, ^28^, ^29^, ^30^ ^]^ Biomimetic nanoparticles, which inherit a rich array of surface antigens from source cells, exhibit multifunctional effects, including targeted delivery, high enrichment, and immune modulation.^[^ ^31^, ^32^, ^33^, ^34^, ^35^ ^]^ Neutrophils, which are key players in inflammatory injury, express surface antigens that bind to adhesion molecules, cells, and chemotactic factors at injury sites.^[^ ^36^, ^37^, ^38^, ^39^ ^]^ Neutrophil membrane‐coated liposomal drug delivery systems effectively bind to chemokines, enabling targeted accumulation at rheumatoid arthritis lesion sites with joint‐specific precision.^[^ ^40^, ^41^ ^]^ Both in vitro and in vivo studies have demonstrated that neutrophil‐derived extracellular vesicles (Neu‐EVs) efficiently encapsulate therapeutic agents and bind to damaged endothelium, alleviating endothelial injury and sepsis‐induced lung damage.^[^ ^39^, ^40^, ^42^ ^]^

**Scheme 1:**
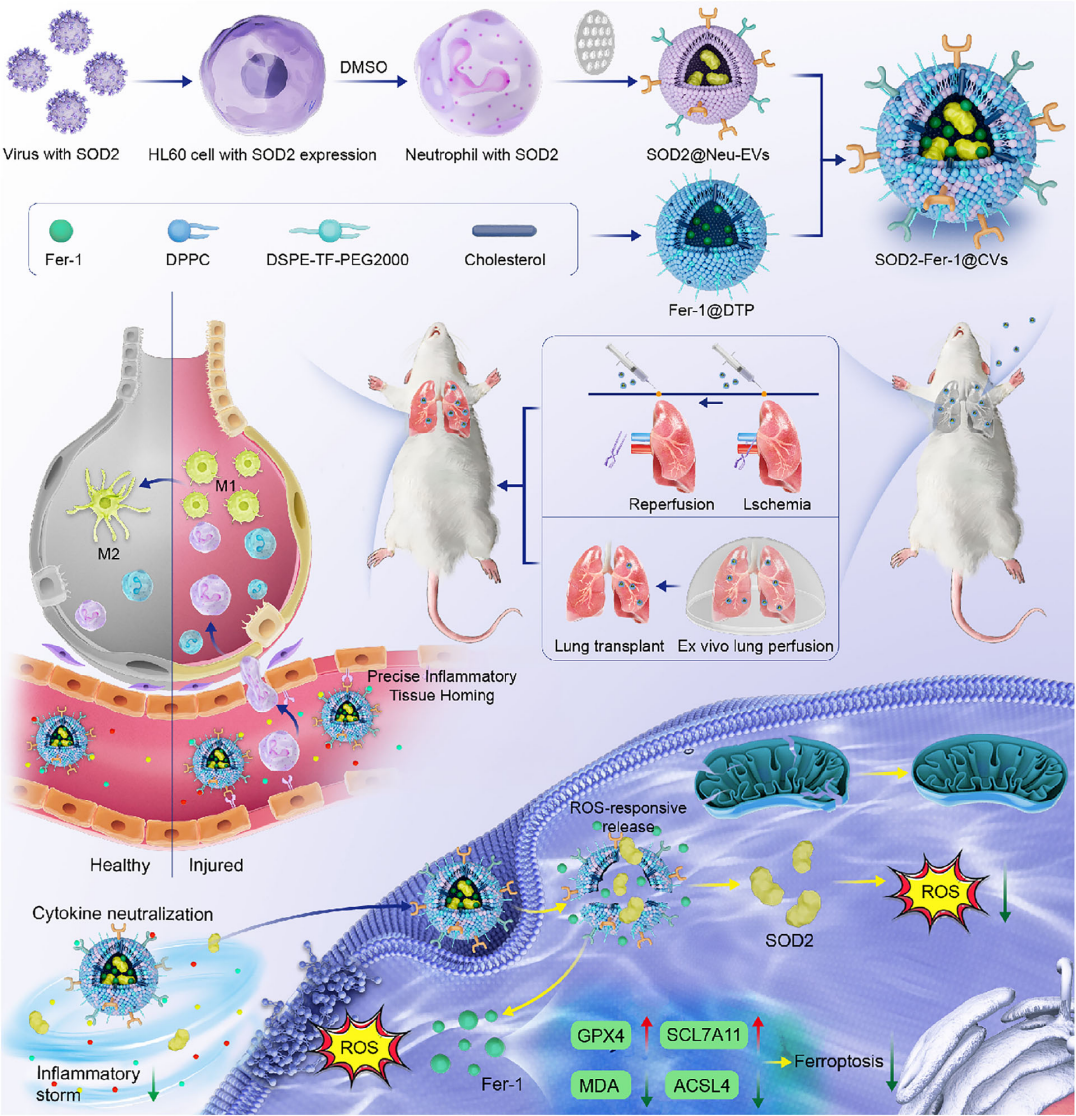
The schematic illustrates engineered neutrophil‐derived ROS‐responsive vesicles containing SOD2 and Ferrostatin‐1 for treating lung transplant‐related primary graft dysfunction.

In this study, we developed neutrophil‐derived ROS‐responsive cellular vesicles engineered with superoxide dismutase 2 (SOD2) and ferrostatin‐1 (SOD2‐Fer‐1@CVs) to address multiple IRI‐related mechanisms, including oxidative stress, ferroptosis, and inflammation, to mitigate lung injury and PGD in LTx (**Scheme** **1**). SOD2‐Fer‐1@CVs are hybrid nano‐systems formed by integrating SOD2‐overexpressing Neu‐EVs with ROS‐responsive liposomes loaded with ferrostatin‐1 (Fer‐1, a potent ferroptosis inhibitor).^[^ ^43^ ^]^ In this study, we aim to develop a multifunctional therapeutic strategy (SOD2‐Fer‐1@CVs) to target key pathological processes involved in lung injury and transplantation. By leveraging the specific targeting capabilities of neutrophil membrane proteins and the combined antioxidant and anti‐ferroptosis effects of SOD2 and Fer‐1, our approach seeks to address inflammation, oxidative stress, and cell death associated with lung ischemia‐reperfusion injury. Integrated into the EVLP platform, this strategy is anticipated to provide a novel and effective means to improve donor lung quality, reduce the incidence of primary graft dysfunction, and ultimately enhance lung transplantation outcomes.

## 2 Results

### 2.1 Fabrication and Characterization of SOD2‐Fer‐1@CVs

**Figure 1:**
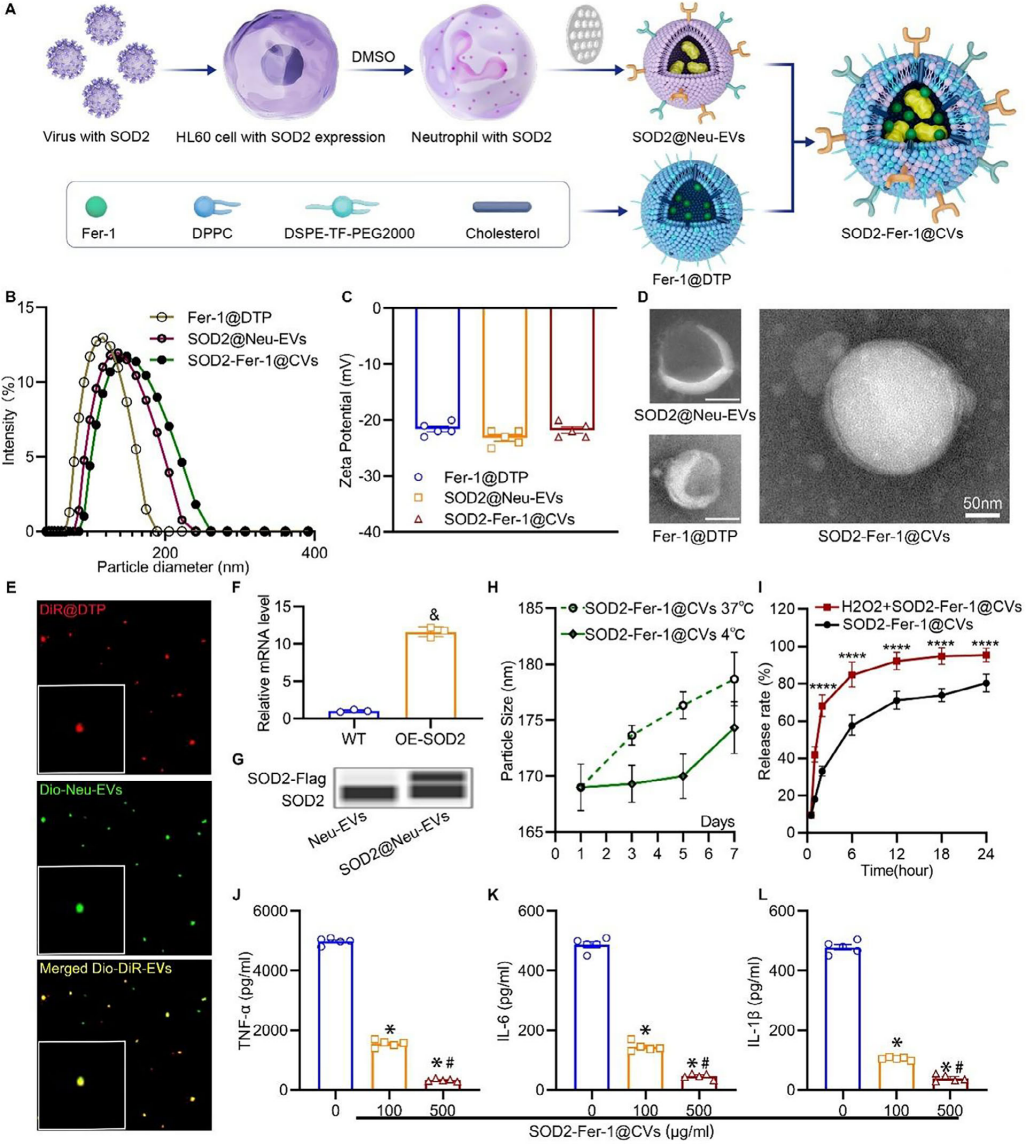
Characterization of SOD2‐Fer‐1@CVs. A) The synthesis process of SOD2‐Fer‐1@CVs. B‐D) Hydrodynamic diameter distributions, zeta potential profiles, and transmission electron microscope image of SOD2@Neu‐EVs, Fer‐1@DTP, and SOD2‐Fer‐1@CVs (scale bar: 50 nm). E) Fluorescent micrograph demonstrating membrane fusion between DiO‐labeled SOD2@Neu‐EVs (green) and DIR‐encapsulated liposomes (DIR@DTP, red) through a polycarbonate membrane, exhibiting merged yellow fluorescence. F) mRNA levels of SOD2 in HL60 cells (wild‐type, WT) and SOD2‐overexpressing HL60 cells (OE‐SOD2) (& versus WT, P<0.05, n = 3). G) Western blot analysis of SOD2 in Neu‐EVs from DMSO‐treated HL60 cells and SOD2@Neu‐EVs from SOD2‐overexpressing HL60 cells following DSMO stimulation. H) Stability assessment of SOD2‐Fer‐1@CVs suspended in 10% FBS solution, showing hydrodynamic diameter variations during 7‐day storage at 4 °C versus 37 °C. I) Cumulative release profiles of Fer‐1 from SOD2‐Fer‐1@CVs under physiological temperature (37 °C) and oxidative stress conditions (50 µM H_2_O_2_) (^****^ versus SOD2‐Fer‐1@CVs, P < 0.001, n = 3). J‐L) Cytokine‐binding capacity of SOD2‐Fer‐1@CVs for TNF‐α, IL‐6 and IL‐1β (^*^ versus 0 µg ml^−1^; # versus 100 µg ml^−1^, P < 0.05, n = 5).

SOD2‐Fer‐1@CVs were synthesized via the membrane fusion of SOD2‐overexpressing neutrophil‐derived extracellular vesicles (SOD2@Neu‐EVs) and ROS‐responsive Fer‐1‐loaded liposomes (Fer‐1@DTP) (**Figure** **1**A). Dynamic light scattering (DLS) showed hydrodynamic diameters of 115 ± 1 nm for Fer‐1@DTP, 121 ± 1 nm for SOD2@Neu‐EVs, and 132± 1 nm for the fused SOD2‐Fer‐1@CVs (Figure 1B). Zeta potential analysis demonstrated robust colloidal stability across all three vesicle formulations (Figure 1C), with absolute values >20 mV. Transmission electron microscopy (TEM) confirmed the spherical morphology and core‐shell structure of SOD2‐Fer‐1@CVs (100–150 nm) (Figure 1D). To validate the fusion efficiency, DiO‐labeled SOD2@Neu‐EVs (green) and DIR‐loaded liposomes (DiR@DTP, red) were observed to exhibit strong fluorescence colocalization using confocal microscopy (Figure 1E). The efficiency of SOD2 overexpression was successfully validated in HL60 cells transfected with a viral vector encoding SOD2 (Figure 1F). The isolated SOD2@Neu‐EVs retained elevated SOD2 levels (Figure 1G). Furthermore, the retention of key neutrophil membrane proteins, including CD11b (also known as integrin αM) and chemokine receptors such as CXC2 and CXCR4, was detected in SOD2@Neu‐EVs and SOD2‐Fer‐1@CVs (Figure S1, Supporting Information). Stability assays in 10% FBS at 37 and 4 °C revealed negligible changes in particle size, supporting robust colloidal stability for biomedical applications (Figure 1H). ROS‐triggered drug release was assessed by incubating SOD2‐Fer‐1@CVs with 50 µM H_2_O_2_, which significantly accelerated Fer‐1 release compared to untreated controls (Figure 1I), confirming the platform's oxidative stress‐responsive payload delivery. SOD2‐Fer‐1@CVs showed concentration‐dependent reduction of pro‐inflammatory cytokine levels (TNF‐α, IL‐6, and IL‐1β) in vitro (Figure 1J–L), demonstrating effective cytokine neutralization.

### 2.2 SOD2‐Fer‐1@CVs Promoted Lung Vascular Endothelial Repair by Suppressing Inflammation and Oxidative Stress

**Figure 2:**
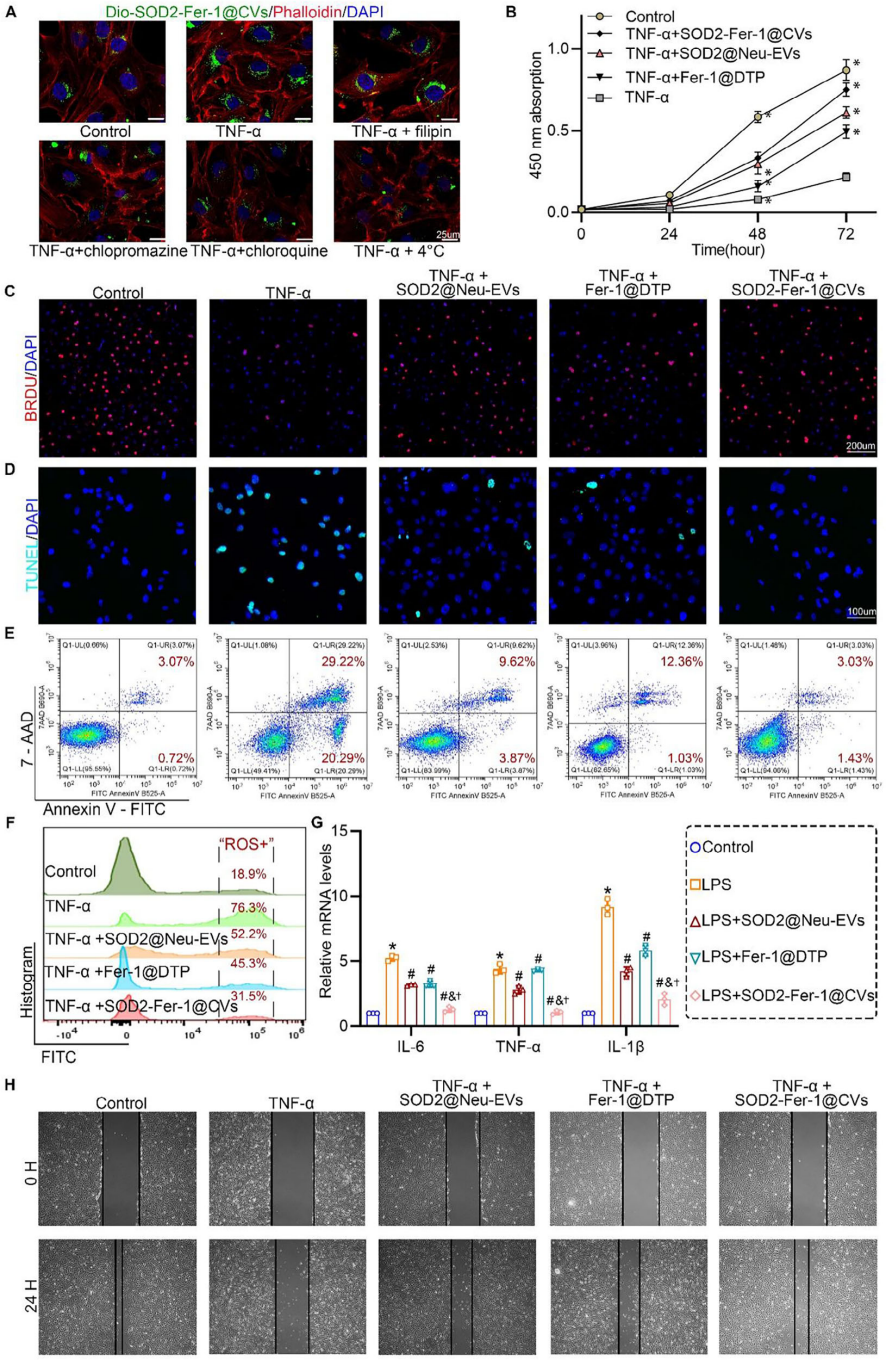
SOD2‐Fer‐1@CVs Ameliorate Inflammatory and Oxidative Stress Injury in Endothelial Cells In Vitro. A) Fluorescence microscopy analysis of DiO‐labeled SOD2‐Fer‐1@CVs (green) uptake by HPMECs pretreated with endocytic pathway inhibitors (chlorpromazine, filipin, chloroquine, and 4 °C incubation) followed by TNF‐α stimulation (scale bar: 25 um). B) CCK‐8 viability assay showing absorbance changes in TNF‐α‐stimulated HPMECs pretreated with SOD2@Neu‐EVs (50 µg ml^−1^), Fer‐1@DTP (Fer‐1: 1 µM), or SOD2‐Fer‐1@CVs (50 µg ml^−1^; Fer‐1: 1 µM) (^*^ versus TNF‐a; P<0.05, n = 5). C‐D) Fluorescence microscopic evaluation of HPMECs proliferation (BrdU incorporation, red, scale bar: 200um) (C) and apoptosis (TUNEL staining, green, scale bar: 100um) (D) following TNF‐α stimulation with indicated treatments. E‐F) Flow cytometric analysis of apoptosis (Annexin V/7‐AAD staining) (E) and intracellular ROS levels (FITC fluorescence) (F) in experimental groups. G) qRT‐PCR quantification of inflammatory cytokines (IL‐6, TNF‐α, IL‐1b) expression in LPS‐stimulated HPMECs pretreated with experimental formulations (^*^ versus Control; # versus LPS; & versus LPS+SOD2@Neu‐EVs; † versus LPS+Fer‐1@DTP, P<0.05, n = 3). H) Scratch wound assay demonstrating migratory capacity of TNF‐α‐stimulated HPMECs with different treatments.

To evaluate targeting efficiency, TNF‐α‐stimulated human pulmonary microvascular endothelial cells (HPMECs) (modeling oxidative stress) showed enhanced uptake of SOD2‐Fer‐1@CVs under high ROS conditions (**Figure** **2**A). Pretreatment with pharmacological inhibitors revealed that low‐temperature conditions (4 °C) most significantly attenuated CVs internalization by suppressing membrane fluidity and energy metabolism (Figure 2A). Additionally, clathrin‐ (chlorpromazine/chloroquine) and caveolae‐dependent (filipin) endocytosis pathways were implicated in the uptake of CVs, as evidenced by the reduced cellular internalization of these inhibitors (Figure 2A). SOD2‐Fer‐1@CVs at concentrations ranging from 0 to 150 µg mL^−1^ had no effect on the proliferation of HPMECs (Figure S2A, Supporting Information). More importantly, SOD2‐Fer‐1@CVs at 50 µg mL^−1^ significantly alleviated the reduction in endothelial cell proliferation induced by TNF‐α (Figure S2B, Supporting Information). TNF‐α induced HPMEC apoptosis and suppressed proliferation, which were markedly reversed by SOD2‐Fer‐1@CVs compared to SOD2@Neu‐EVs or Fer‐1@DTP alone (Figure 2B–E). The hybrid platform also effectively neutralized TNF‐α‐triggered ROS overproduction (Figure 2F). In lipopolysaccharide (LPS)‐challenged HPMECs, SOD2‐Fer‐1@CVs significantly downregulated the transcription of pro‐inflammatory cytokines (IL‐6, TNF‐α, and IL‐1β) (Figure 2G). Scratch assays further demonstrated that SOD2‐Fer‐1@CVs uniquely restored endothelial migration capacity impaired by TNF‐α, highlighting their synergistic repair potential (Figure 2H; Figure S3, Supporting Information).

### 2.3 SOD2‐Fer‐1@CVs Inhibited Ferroptosis to Alleviate Endothelial Dysfunction

**Figure 3:**
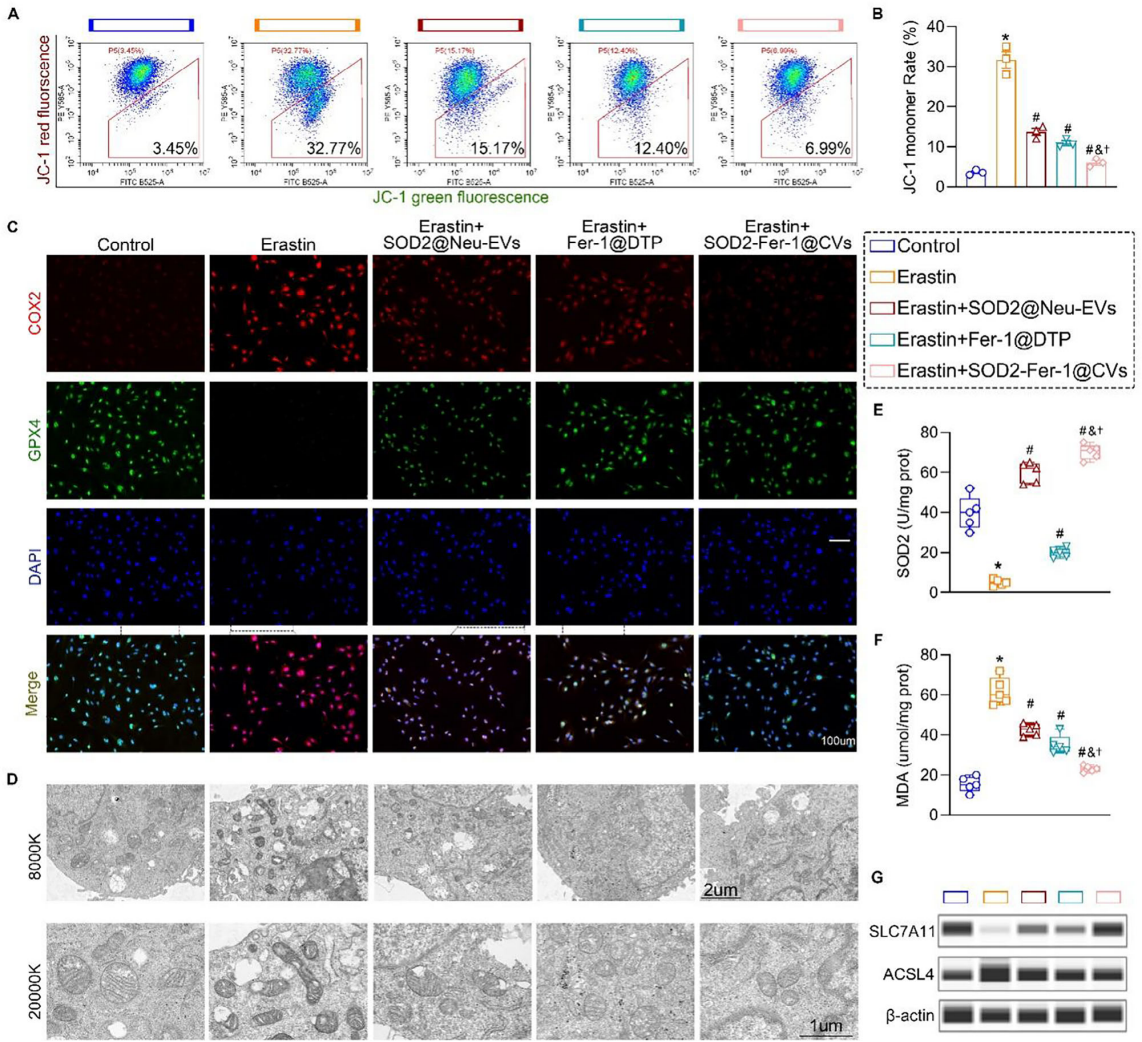
SOD2‐Fer‐1@CVs Attenuate Ferroptosis and Mitochondrial Dysfunction in HPMECs. A‐B) JC‐1 fluorescence intensity analysis demonstrating Erastin‐induced mitochondrial membrane potential (ΔΨm) collapse in HPMECs and therapeutic efficacy of SOD2@Neu‐EVs (50 µg ml^−1^), Fer‐1@DTP (Fer‐1: 1 µM), and SOD2‐Fer‐1@CVs (50 µg ml^−1^; Fer‐1: 1 µM) in maintaining ΔΨm integrity. C) Immunofluorescence imaging of ferroptosis biomarkers GPX4 (green) and COX‐2 (red) in Erastin‐challenged HPMECs pretreated with experimental formulations, with nuclear counterstaining using DAPI (blue) (Scale bar: 100um). D) Representative transmission electron microscopy (TEM) images illustrating mitochondrial ultrastructural changes under specified experimental conditions (Scale bar, up: 2um; Scale bar, down: 1um). E‐F) Quantitative analysis of superoxide dismutase 2 (SOD2) enzymatic activity (E) and lipid peroxidation marker malondialdehyde (MDA) levels (F) via ELISA in Erastin‐treated HPMECs. G) Western blot profiling of ferroptosis‐regulatory proteins (SLC7A11 and ACSL4) expression patterns in intervention groups. ^*^ versus Control; # versus Erastin; & versus Erastin+SOD2@Neu‐EVs; † versus Erastin+Fer‐1@DTP, P< 0.05.

In erastin‐stimulated HPMECs, JC‐1 flow cytometry revealed a marked reduction in the mitochondrial membrane potential (increased JC‐1 monomer ratio) and mitochondrial dysfunction (**Figure** **3**A). Pretreatment with SOD2‐Fer‐1@CVs restored mitochondrial polarity, as evidenced by elevated levels of JC‐1 aggregates, and preserved mitochondrial homeostasis (Figure 3A,B). Erastin downregulated glutathione peroxidase 4 (GPX4) and upregulated cyclooxygenase‐2 (COX2), which are key ferroptosis markers, while SOD2‐Fer‐1@CVs pretreatment effectively reversed these trends, restoring GPX4 expression and suppressing COX2 overexpression (Figure 3C). Transmission electron microscopy (TEM) corroborated these findings; erastin triggered mitochondrial swelling, increased membrane density, and cristae loss, whereas SOD2‐Fer‐1@CVs pretreatment attenuated these alterations and restored mitochondrial morphology (Figure 3D). Erastin suppressed the endogenous *SOD2* expression, which was rescued by treatment with SOD2@Neu‐EVs and SOD2‐Fer‐1@CVs (Figure 3E). SOD2‐Fer‐1@CVs mitigated erastin‐induced lipid peroxidation, as indicated by elevated malondialdehyde (MDA) levels (Figure 3F). Western blotting further demonstrated that SOD2‐Fer‐1@CVs inhibited ferroptosis by preventing the erastin‐mediated downregulation of the amino acid transport system xc‐ (*SLC7A11)* and suppressing the upregulation of *ACSL4* (Figure 3G). These results collectively established the dual role of SOD2‐Fer‐1@CVs in counteracting ferroptosis via mitochondrial protection, lipid peroxidation inhibition, and regulation of ferroptosis‐related pathways.

### 2.4 SOD2‐Fer‐1@ CVs Drove Macrophage Repolarization from M1 to M2 Phenotype and Attenuate Endothelial Injury

**Figure 4:**
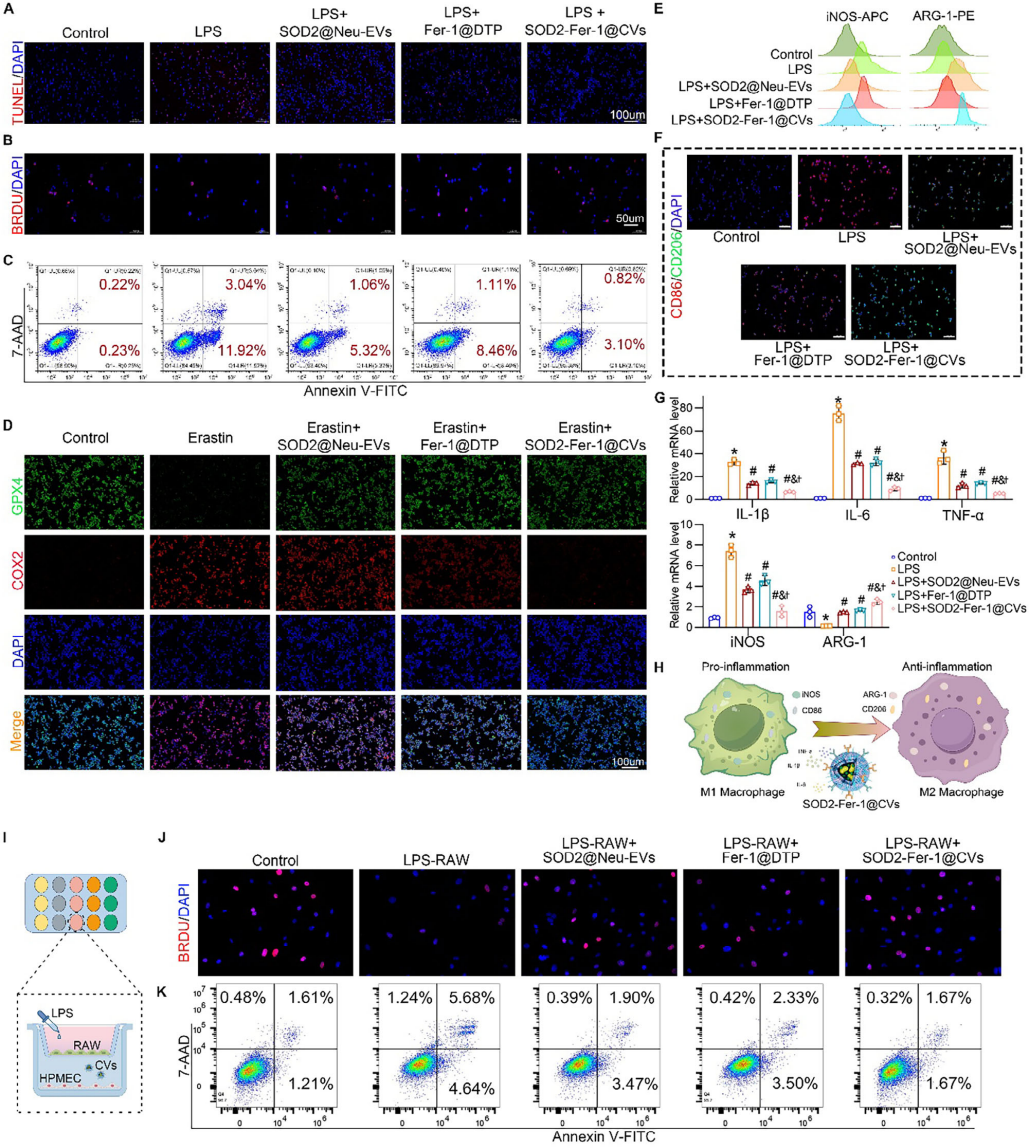
SOD2‐Fer‐1@CVs Facilitate M1‐to‐M2 Macrophage Polarization and Modulate Endothelial Crosstalk In Vitro. A‐B) Fluorescence microscopy evaluation of LPS‐stimulated RAW264.7 macrophages pretreated with SOD2@Neu‐EVs (50 µg ml^−1^), Fer‐1@DTP (Fer‐1: 1 µM), or SOD2‐Fer‐1@CVs (50 µg ml^−1^; Fer‐1: 1 µM), showing apoptotic signatures (TUNEL staining, green, scale bar:100um) (A) and proliferative activity (BrdU incorporation, red, scale bar:50um) (B). C) Flow cytometric quantification of apoptosis using Annexin V/7‐AAD dual staining. D) Immunofluorescence analysis of ferroptosis biomarkers GPX4 (green) and COX‐2 (red) expression with nuclear counterstaining (DAPI) in corresponding treatment groups (scale bar: 100 µm). E) Flow cytometric analysis of LPS‐induced RAW264.7 macrophages co‐stained with APC‐conjugated anti‐iNOS (M1 marker) and PE‐conjugated anti‐ARG‐1 (M2 marker), demonstrating polarization modulation by SOD2@Neu‐EVs, Fer‐1@DTP, and SOD2‐Fer‐1@CVs pretreatment. F) Fluorescence microscopy visualization of CD86 (red, M1 marker) and CD206 (green, M2 marker) expression patterns in treatment groups, counterstained with DAPI (blue) for nuclear localization (Scale bar: 100um). G) qRT‐PCR quantification of pro‐inflammatory cytokines (IL‐6, TNF‐α, IL‐1β) and polarization‐related genes (iNOS and ARG‐1) in LPS‐stimulated RAW264.7 macrophages pretreated with experimental formulations. H) Integrated assessment of macrophage functional polarization. I‐K) Transwell co‐culture system of LPS‐polarized M1 macrophages and HPMECs (I), Fluorescence microscopic assessment of HPMEC proliferation (BrdU, red; DAPI nuclear stain) under macrophage‐mediated inflammatory stress (J), and flow cytometric profiling of endothelial apoptosis using Annexin V‐FITC/PI dual labeling (K). ^*^ versus Control; # versus LPS; & versus LPS+SOD2@Neu‐EVs; † versus LPS+Fer‐1@DTP, P < 0.05, n = 3.

BrdU and TUNEL assays revealed that SOD2‐Fer‐1@CVs significantly enhanced macrophage proliferation and reduced apoptosis (**Figure** **4**A–C). Immunofluorescence analysis demonstrated that SOD2‐Fer‐1@CVs suppressed erastin‐induced ferroptosis in macrophages, as shown by the restoration of *GPX4* expression and reduction in *COX2* signals, mirroring the protective effects observed in endothelial cells (Figure 4D). For macrophage polarization studies, LPS‐stimulated RAW264.7 cells were pre‐polarized toward the M1 phenotype, characterized by upregulated inducible nitric oxide synthase (iNOS) and downregulated arginase‐1 (ARG‐1) levels (Figure 4E). Flow cytometry revealed that SOD2‐Fer‐1@ CVs significantly reduced *iNOS* fluorescence intensity while enhancing *ARG‐1* signals, indicating M1‐to‐M2 repolarization (Figure 4E). Immunofluorescence analysis further confirmed the superior efficacy of SOD2‐Fer‐1@CVs compared with those of individual SOD2@Neu‐EVs or Fer‐1@DTP, demonstrating obvious downregulation of the M1 marker, CD86, and concurrent upregulation of the M2 marker, CD206 (Figure 4F). Consistent with these findings, quantitative reverse transcription‐polymerase chain reaction (qRT‐PCR) analysis showed that SOD2‐Fer‐1@CVs suppressed LPS‐induced transcription of pro‐inflammatory mediators (*IL‐1β*, *IL‐6*, *TNF‐α*, and *iNOS*) while promoting *ARG‐1* expression (Figure 4G). Collectively, these data underscore the capacity of SOD2‐Fer‐1@CVs to reprogram macrophage plasticity by reducing and neutralizing inflammatory cytokines (Figure 1J–L) and driving phenotypic switching toward anti‐inflammatory M2 states (Figure 4H). To evaluate M1 macrophage‐driven endothelial injury, a co‐culture system of RAW264.7 macrophages and HPMECs was established (Figure 4I). Flow cytometry showed that LPS‐polarized M1 macrophages exacerbated endothelial apoptosis and inhibited endothelial proliferation, whereas pretreatment with SOD2‐Fer‐1@CVs markedly reduced these harmful effects (Figure 4J,K). These findings confirmed that SOD2‐Fer‐1@CVs not only inhibit M1 activation but also inhibit their capacity to induce endothelial dysfunction, underscoring their therapeutic potential in modulating macrophage‐endothelial crosstalk.

### 2.5 SOD2‐Fer‐1@CVs Attenuated Left Lung IRI in Rats

**Figure 5:**
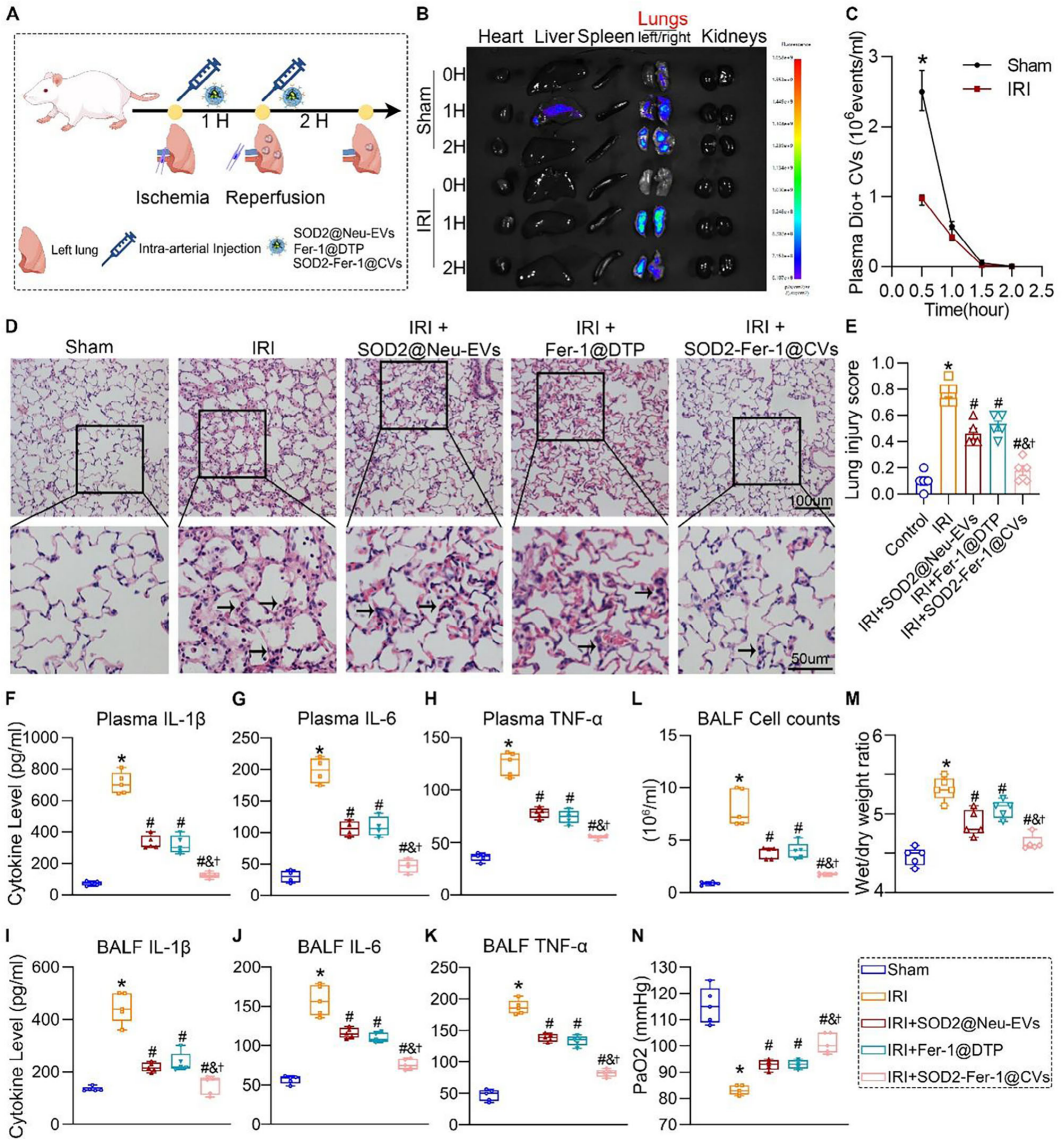
SOD2‐Fer‐1@CVs Mitigate Ischemia‐Reperfusion‐Induced Acute Lung Injury in Rats. A) Experimental design schematic: Left pulmonary hilum clamping (1 h ischemia) followed by reperfusion (2 h) establishing an IRI‐induced ALI model, with therapeutic interventions administered via intra‐iliac arterial injection of SOD2@Neu‐EVs (0.5 mg kg^−1^), Fer‐1@DTP (Fer‐1: 0.03 mg kg^−1^, or SOD2‐Fer‐1@CVs (0.5 mg kg^−1^; Fer‐1: 0.03 mg kg^−1^) pre‐ischemia and post‐reperfusion. B‐C) Biodistribution analysis: (B) Ex vivo fluorescence imaging of DiR‐labeled SOD2‐Fer‐1@CVs in major organs; (C) Nanoscale flow cytometric quantification of Dio‐labeled SOD2‐Fer‐1@CVs in peripheral blood. D‐E) Histopathological evaluation: (D) Representative H&E‐stained lung sections showing inflammatory infiltrates (black arrows) (Up scale bar: 100um, Down scale bar: 50um); (E) Semi‐quantitative lung injury scoring based on histomorphology criteria. F‐K) Functional assessments: ELISA quantification of pro‐inflammatory cytokines (IL‐1β, IL‐6, TNF‐α) in plasma (F‐H) and bronchoalveolar lavage fluid (BALF) (I‐K); (L) Total cell count in BALF; (M) Pulmonary edema index calculated by wet/dry weight ratio; (N) Arterial oxygen partial pressure (PaO_2_) measured by automated blood gas analysis. ^*^ versus Sham; # versus IRI; & versus IRI+SOD2@Neu‐EVs; † versus IRI+Fer‐1@DTP, P < 0.05.

We established a rat model of IRI‐induced lung injury to investigate the role of CVs (**Figure** **5**A). DIR‐labeled SOD2‐Fer‐1@CVs administered via the iliac artery exhibited enhanced accumulation in ischemic lung tissue compared to that in sham‐operated controls, as evidenced by in vivo fluorescence imaging (Figure 5B). Pharmacokinetic analysis revealed a significant reduction in circulating Dio‐labeled SOD2‐Fer‐1@CVs post‐IRI, underscoring their injury‐targeting specificity (Figure 5C). Histopathological evaluation [hematoxylin‐eosin (H&E) staining] demonstrated that IRI induced severe lung injury, which was characterized by inflammatory cell infiltration, alveolar septal thickening, and interstitial edema (Figure 5D). SOD2‐Fer‐1@CVs (0.5 mg kg^−1^) significantly mitigated these pathological alterations, outperforming SOD2@Neu‐EVs and Fer‐1@DTP in reducing the lung injury scores (Figure 5E; Figure S4, Supporting Information). Furthermore, SOD2‐Fer‐1@CVs suppressed inflammatory cytokine levels in the plasma and bronchoalveolar lavage fluid (BALF), decreased inflammatory cell infiltration in the BALF, and lowered the lung wet/dry weight ratio, collectively alleviating IRI‐induced hypoxemia (Figure 5F–N). These findings demonstrated the therapeutic efficacy of SOD2‐Fer‐1@CVs in attenuating lung ischemia‐reperfusion injury via targeted anti‐inflammatory mechanisms.

### 2.6 SOD2‐Fer‐1@CVs Alleviated Lung Injury by Suppressing Oxidative Stress and Ferroptosis

**Figure 6:**
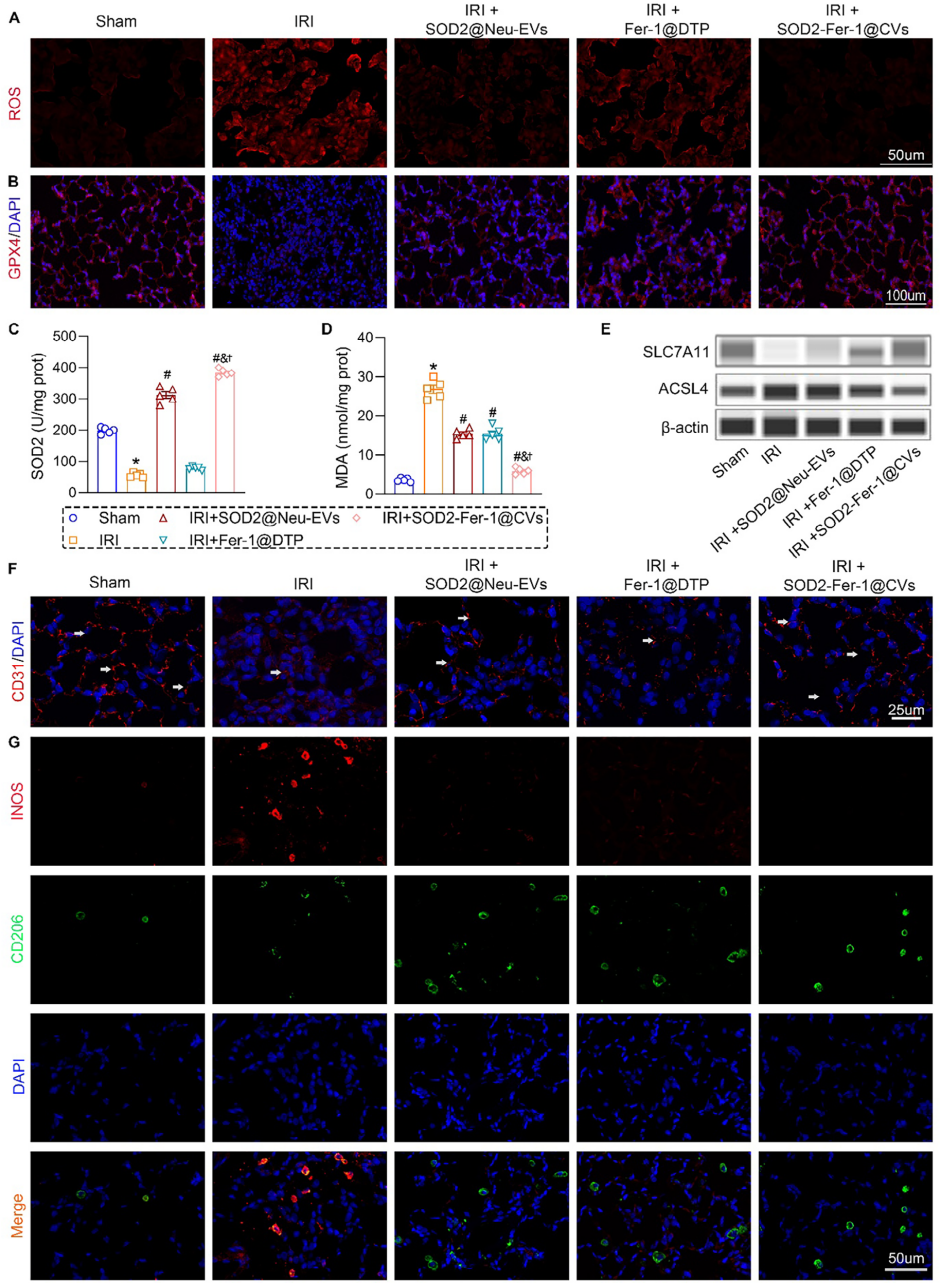
SOD2‐Fer‐1@CVs Ameliorate IRI‐Induced ALI via Coordinated Suppression of Inflammation, Oxidative Stress, and Ferroptosis. A‐B) In situ redox profiling: (A) ROS levels in lung sections from IRI rat models with therapeutic interventions including SOD2@Neu‐EVs (0.5 mg kg^−1^), Fer‐1@DTP (Fer‐1: 0.03 mg kg^−1^, or SOD2‐Fer‐1@CVs (0.5 mg kg^−1^; Fer‐1: 0.03 mg kg^−1^) (Scale bar: 50um). (B) Immunohistochemical analysis of ferroptosis marker GPX4 (red) expression in pulmonary parenchyma (Scale bar: 100 µm). C‐D) Biochemical characterization: ELISA quantification of SOD2 enzymatic activity (C) and lipid peroxidation product MDA levels (D) in lung tissue homogenates. E) Western blot analysis of ferroptosis‐regulatory proteins (SLC7A11 and ACSL4) expression in lung tissues. F) Confocal microscopy visualization of vascular endothelium integrity via CD31 immunofluorescence (endothelial marker, red; DAPI nuclear counterstain, blue, scale bar: 25um). G) Dual immunofluorescence co‐staining of macrophage polarization markers (iNOS[+] M1: red; CD206[+] M2: green) in alveolar regions, with DAPI‐labeled nuclei (blue) (Scale bar:50um). ^*^ versus Sham; # versus IRI; & versus IRI+SOD2@Neu‐EVs; † versus IRI+Fer‐1@DTP, P < 0.05, n = 5.

IRI triggered profound oxidative stress in rat lungs, as evidenced by elevated ROS levels compared to those in sham controls (**Figure** **6**A). Furthermore, IRI induced ferroptosis signaling by downregulating of antioxidant regulators (GPX4 and SOD2) and increasing levels of lipid peroxidation marker, MDA (Figure 6B–D). Treatment with SOD2‐Fer‐1@CVs significantly attenuated these pathological changes (Figure 6A–D). Western blot analysis confirmed IRI‐mediated ferroptosis via ACSL4 upregulation, coupled with SLC7A11 suppression, which was reversed by SOD2‐Fer‐1@CVs (Figure 6E). Immunofluorescence analysis revealed that SOD2‐Fer‐1@CVs prevented the IRI‐induced downregulation of the endothelial marker, CD31, and preserved microvascular barrier integrity (Figure 6F). Similar to in vitro findings, IRI promoted M1 macrophage polarization (increased iNOS level) in lung tissue, whereas SOD2‐Fer‐1@CVs shifted macrophages toward the M2 phenotype (elevated CD206), further attenuating inflammation (Figure 6G). These results demonstrated the therapeutic mechanism of SOD2‐Fer‐1@CVs in mitigating lung injury via the dual modulation of oxidative stress and ferroptosis.

### 2.7 SOD2‐Fer‐1@CVs Combined with EVLP Reduced Lung Injury in LTx

**Figure 7:**
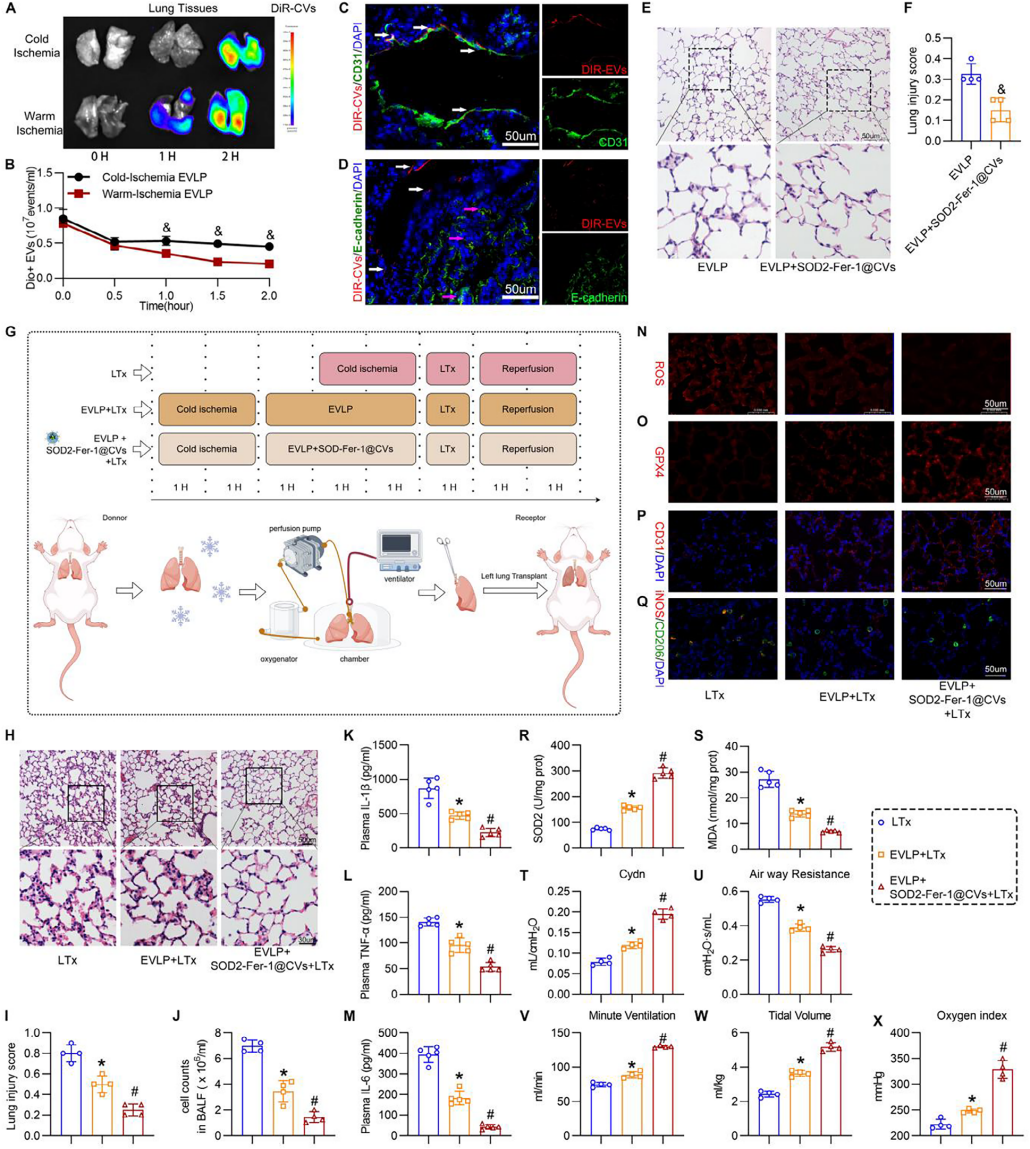
SOD2‐Fer‐1@CVs Enhance Pulmonary Graft Recovery via Ex Vivo Lung Perfusion (EVLP) to Attenuate lung IRI after LTx. A‐B) Biodynamic monitoring during EVLP: (A) Time‐lapsed near‐infrared imaging of DiR‐labeled SOD2‐Fer‐1@CVs in cold/warm ischemic donor lungs; (B) Nanoscale flow cytometric tracking of DiO‐labeled CVs in perfusion circuit (& versus Warm‐Ischemia EVLP, P<0.05, n = 3). C‐D) Immunofluorescence co‐localization analysis of CD31⁺ endothelial cells (green) and DiR‐labeled CVs (red) (C) and the localization analysis of E‐cadherin⁺ epithelial cells (green) and DiR‐labeled CVs (red) (D) in graft tissues (DAPI nuclear counterstain, blue) (Scale bar: 50 µm). E‐F) Comparative histopathological evaluation of EVLP lungs with/without SOD2‐Fer‐1@CVs (0.5 mg kg^−1^; Fer‐1: 0.03 mg kg^−1^) intervention (H&E) before LTx (E) and the lung injury score (F) (& versus EVLP, P<0.05, n = 4). G) Schematic of therapeutic protocol integrating SOD2‐Fer‐1@CVs with EVLP for lung repair in a rat lung transplantation (LTx)‐induced lung injury model. H‐S) Post‐transplant assessment: (H) Representative H&E sections from LTx, EVLP+LTx, and EVLP+SOD2‐Fer‐1@CVs (0.5 mg kg^−1^; Fer‐1: 0.03 mg kg^−1^)+LTx groups; (I) the lung injury socre based on H&E staining; (J) BALF cell count quantification; (K‐M) Plasma IL‐1β/IL‐6/TNF‐α levels; N‐Q) Mechanistic profiling (Scale bar: 50um): (N) pulmonary ROS quantification (red); (O) GPX4 immunofluorescence (ferroptosis marker, red); (P) CD31⁺ endothelial integrity (red); (Q) Dual‐polarization mapping of alveolar macrophages (iNOS⁺ M1: red; CD206⁺ M2: green; DAPI: blue); (R‐S) Pulmonary MDA/SOD2 levels. T‐X) Pulmonary function analysis: dynamic compliance (Cydn) (T); Airway pressure (U), Minute Ventilation (V), Tide Volume (W), and Oxygen index (X). ^*^ versus LTx; # versus EVLP+LTx, P < 0.05, for I‐M and R‐X.

EVLP is as a critical platform for donor lung evaluation and injury repair. First, DIR‐ or DiO‐labeled SOD2‐Fer‐1@CVs were infused into the EVLP circuit without LTx, revealing significantly increased DiR‐labeled CVs uptake in injured lungs (warm ischemia) compared to that in controls (cold ischemia), along with a reduction in Dio‐labeled CVs concentration in the perfusate, confirming injury‐targeted delivery (**Figure** **7**A,B). Immunofluorescence analysis of lung sections demonstrated strong co‐localization of DiR‐labeled CVs (red) with CD31 (endothelial marker) but minimal overlap with E‐cadherin (epithelial marker), indicating preferential targeting of the lung vascular endothelium during EVLP (Figure 7C,D). In vitro studies further demonstrated that rat pulmonary microvascular endothelial cells can efficiently take up SOD2‐Fer‐1@CVs (Figure S5, Supporting Information). Without LTx, compared to EVLP alone, SOD2‐Fer‐1@CVs combined with EVLP markedly reduced inflammatory infiltration and edema (Figure 7E,F), suppressed perfusate TNF‐α and IL‐6 levels, and attenuated oxidative stress (Figure S6A–C, Supporting Information). This was accompanied by preserved endothelial integrity (CD31 expression) and enhanced M2 macrophage polarization in the treated lungs (Figure S6D,E, Supporting Information). Collectively, these results established that SOD2‐Fer‐1@CVs synergize with EVLP to inhibit reperfusion injury.

Figure 7G shows the design of the experimental group for evaluating the therapeutic efficacy of the combination of SOD2‐Fer‐1@CVs and EVLP perfusion in decreasing post‐transplant lung injury. The results demonstrated that LTx induced severe pulmonary injury characterized by inflammatory cell infiltration, alveolar protein exudation, and interstitial edema (Figure 7H). Compared to EVLP + LTx alone, the combination of EVLP with SOD2‐Fer‐1@CVs in LTx exhibited superior lung repair capabilities, including reduced inflammatory cell infiltration in lung tissues and BALF, decreased interstitial edema, and lower lung injury scores (Figure 7H–J) as well as downregulation of plasma cytokine expression (IL‐1β, TNF‐α, and IL‐6) (Figure 7K–M). SOD2‐Fer‐1@CVs, combined with the EVLP, reduced ROS production and alleviated oxidative stress (Figure 7N). SOD2‐Fer‐1@CVs also improved lung vascular endothelial cell function and promoted macrophage M2 polarization, along with inhibition of ferroptosis (decreased GPX4/MDA and elevated SOD2 levels) and anti‐inflammatory and antioxidant stress effects (Figure 7O–S). Assessment of pulmonary function demonstrated that SOD2‐Fer‐1@CVs, combined with EVLP, significantly improved post‐transplantation lung compliance, reduced airway pressure, and restored minute ventilation with tidal volume, resulting in enhanced pulmonary tissue oxygenation capacity (Figure 7T–X).

### 2.8 SOD2‐Fer‐1@CVs Ameliorated Pulmonary Injury in IRI and EVLP Models via Coordinated Suppression of Inflammatory Signaling, Oxidative Stress, and Cell Death Pathways Coupled with Enhanced Energy Metabolism

To systematically investigate the molecular mechanisms underlying the therapeutic interventions, we performed RNA sequencing on pulmonary tissues from IRI cohorts, IRI (n = 4) and IRI treated with SOD2‐Fer‐1@CVs (n = 4) (Figure S7A,B, Supporting Information), and EVLP cohorts, EVLP (n = 4) and EVLP treated with SOD2‐Fer‐1@CVs (n = 4) (Figure S7C,D, Supporting Information). Gene Set Enrichment analysis (GSEA) analysis with network diagrams showed that compared to the control group, SOD2‐Fer‐1@CVs stimulation significantly reduced pathways linked to inflammation (immune cell activation, granulocyte migration and chemotaxis), oxidative stress (superoxide anion oxygen), and cell death (apoptotic pathway receptors), while strongly increasing energy metabolism‐related pathways (transmembrane transport) (Figure S8, Supporting Information). The GSEA results of lung perfusion with SOD2‐Fer‐1@CVs combined with EVLP showed that SOD2‐Fer‐1@CVs significantly boosted energy metabolism (condensed chromosome cycle) while reducing inflammation pathways (monocyte leukocyte differentiation) and cell death compared with the EVLP group (Figure S9, Supporting Information).

Furthermore, we used qRT‐PCR to validate the sequencing results. In the IRI model (Figure S10A–D, Supporting Information), SOD2‐Fer‐1@CVs increased the expression of antioxidant enzymes glutathione peroxidase 2 (GPX2), glutathione peroxidase 7 (GPX7), and nitric oxide synthase 3 (NOS3), helping to reduce oxidative stress. SOD2‐Fer‐1@CVs also downregulated phospholipase A2 group V (PLA2G5), which is involved in inflammation, and inhibited Caspase 8 and increased B‐cell lymphoma 2 like 2 (BCL2L2), supporting cell survival and reducing cell death. In addition, SOD2‐Fer‐1@CVs suppressed pyruvate dehydrogenase kinase 3 (PDK3) to promote energy metabolism.

In the EVLP combined with LTx model, SOD2‐Fer‐1@CVs inhibited nuclear factor kappa B subunit 2 (NF‐κB2) and reduced C‐C motif chemokine ligand 3 (CCL3) and C‐C chemokine receptor type 5 (CCR5) expression, thereby decreasing inflammation. They also promoted autophagy‐related 5 (ATG5) to reduce cell death and increased glycerol‐3‐phosphate dehydrogenase 1 (GPD1) expression to support mitochondrial energy metabolism (Figure S10E–G, Supporting Information).

### 2.9 Biosafety Assessment of SOD2‐Fer‐1@CVs

Systemic biosafety evaluation demonstrated preserved histoarchitecture in all major organs (heart, liver, spleen, kidneys, and lungs) of SOD2‐Fer‐1@CVs‐treated rats, showing no evidence of inflammatory infiltration or necrosis compared to sham controls (Figure S11A, Supporting Information). Levels of the markers of hepatic function remained within physiological ranges (ALT and AST) with comparable renal clearance profiles (BUN and CREA) (Figure S11B–E, Supporting Information). Hemocompatibility assessment revealed minimal hemolytic potential (Figure S11F, Supporting Information). These data collectively confirmed the translational viability of this nanotherapeutic platform based on multi‐organ safety validation.

## 3 Discussion

This study demonstrated that SOD2‐Fer‐1@CVs, a hybrid nanoplatform engineered by fusing of Neu‐EVs and ROS‐responsive liposomes, synergistically mitigated pulmonary IRI via coordinated modulation of inflammatory signaling, oxidative stress, and ferroptosis. By combining the intrinsic anti‐inflammatory properties of Neu‐EVs (e.g., cytokine scavenging), SOD2‐mediated ROS neutralization, and Fer‐1‐dependent ferroptosis inhibition, SOD2‐Fer‐1@CVs outperformed single‐component therapies in restoring endothelial integrity, repolarizing macrophages toward M2 phenotypes, and preserving mitochondrial function across both in vitro and in vivo models. Crucially, the integration of EVLP with SOD2‐Fer‐1@CVs not only enhanced donor lung repair but also reduced PGD incidence in transplantation settings, reflecting its translational potential.

Thus, ROS‐responsive liposomes represent a breakthrough in targeted drug delivery. Their thioketal linker enables Fer‐1@DTP to precisely release Fer‐1, specifically in oxidative environments, such as ischemic lungs, minimizing off‐target effects, a feature confirmed by our H_2_O_2_‐triggered release experiments. ROS‐responsive drug delivery platforms leverage pathological oxidative stress to enable targeted drug release, exemplified by thioketal‐based nanoparticles (BA@DPTP) that suppress NF‐κB/NLRP3‐mediated pyroptosis and endothelial barrier repair in lung injury,^[^ ^44^ ^]^ and fusion nanovesicles (FNVs@RAPA) that reprogram Ly6C^+^Ly6G^−^ macrophages to alleviate cardiac transplant rejection.^[^ ^45^ ^]^ Like our study, recent advancements have highlighted multifunctional integration, combining antioxidants, immunomodulators, and biomimetic targeting strategies (e.g., mesenchymal stem cell membranes) to enhance spatiotemporal precision across organs, including the lungs and heart.^[^ ^46^, ^47^ ^]^ Neu‐EVs naturally target inflamed blood vessels owing to inherited membrane proteins, as shown by DiR imaging in the lung injury models in this study. Neu‐EVs demonstrate exceptional promise as drug delivery vehicles, leveraging their innate biocompatibility, low immunogenicity, and intrinsic targeting capabilities—such as integrin β2‐ICAM‐1 interactions for inflamed lung endothelial targeting—to deliver therapeutics such as piceatannol methylprednisolone sodium, effectively suppressing cytokine release, bacterial proliferation, and neutrophil infiltration in lung injury models.^[^ ^40^, ^42^, ^48^, ^49^ ^]^ By combining this inflammation‐homing ability with ROS‐triggered drug release, SOD2‐Fer‐1@CVs exemplify next‐generation “smart” nanotherapeutics. Besides, liposomes are widely used clinically, with at least 19 liposomal drugs approved by the FDA and EMA, confirming their safety profile.^[^ ^50^ ^]^ EVs are attractive drug delivery vehicles in part because they can reduce the side effects of exogenous agents. Large‐scale studies using human HEK293‐derived EVs have shown that repeated injections in mice did not alter the immune response.^[^ ^51^ ^]^ Compared to cell therapy, EV‐based therapies have much lower immunogenicity.^[^ ^52^ ^]^ These provide the biosafety support for SOD2‐Fer‐1@CVs.

IRI is the central pathological driver of PGD following lung transplantation and is characterized by excessive inflammation, oxidative stress, and cell death, which collectively exacerbate pulmonary dysfunction.^[^ ^6^, ^8^ ^]^ These pathophysiological processes are not only interconnected but also synergistically amplify tissue damage, rendering conventional single‐target therapies inadequate. The current clinical management of IRI or PGD lacks effective pharmacological interventions, underscoring the urgent need for multifaceted therapeutic strategies. Our study introduced SOD2‐Fer‐1@CVs as a hybrid nanoplatform designed to address the complexity of IRI‐driven PGD, combining the intrinsic targeting capabilities of Neu‐EVs, which home to the inflamed pulmonary vasculature, with ROS‐responsive drug delivery to ensure the precise release of the ferroptosis inhibitor, Fer‐1, in oxidative microenvironments. Additionally, SOD2 neutralizes ROS, mitigates oxidative stress, and preserves mitochondrial function. Collectively, these features enable SOD2‐Fer‐1@CVs to suppress inflammation, inhibit ferroptosis, and restore bioenergetic homeostasis, thereby addressing the multifactorial nature of IRI. Recent advancements in nanomedicine have highlighted the potential of hybrid systems in treating complex diseases by simultaneously targeting multiple pathological processes. For instance, studies have demonstrated that multifunctional nanoparticles can alleviate sepsis,^[^ ^53^ ^]^ retinal diseases,^[^ ^54^ ^]^ and arthritis^[^ ^55^ ^]^ by integrating anti‐inflammatory, antioxidant, and cell death‐inhibiting properties. By combining these advanced therapeutic principles, SOD2‐Fer‐1@CVs provide a clinically viable solution for overcoming the limitations of conventional single‐mechanism therapies. This integrated approach not only addresses the multifactorial nature of IRI, but also paves the way for the effective treatment of PGD in clinical settings.

EVLP has revolutionized donor lung reconditioning by enabling extended preservation, functional assessment, and targeted repair of marginal lungs.^[^ ^17^, ^18^, ^20^ ^]^ Our small‐animal EVLP model replicated clinical protocols, demonstrating its efficacy in preserving endothelial integrity and reducing inflammation. Transcriptomics analysis revealed sharing of IRI mechanisms during EVLP and transplantation: TLR/MYD88‐mediated inflammation, oxidative stress, and apoptosis.^[^ ^14^ ^]^ Recent advancements in sequencing technologies, particularly single‐cell RNA sequencing, have significantly enhanced our understanding of the molecular alterations that occur during EVLP and transplantation. Extended EVLP in marginal human donor lungs has been shown to markedly upregulate inflammatory pathways and apoptosis‐related signals within endothelial cells and alveolar macrophages, accompanied by progressive mitochondrial dysfunction.^[^ ^21^, ^56^ ^]^ Clinical correlative analyses have demonstrated that EVLP‐associated inflammatory priming directly correlates with adverse post‐transplant outcomes, including elevated PGD incidence.^[^ ^57^ ^]^ Metabolomic profiling further revealed critical energy substrate depletion, accelerated proteolysis, and nucleoside catabolism during prolonged EVLP, with concomitant accumulation of cytotoxic metabolites.^[^ ^58^, ^59^ ^]^ SOD2‐Fer‐1@CVs synergistically targeted these pathways via (1) ROS‐triggered Fer‐1 release, (2) SOD2‐driven redox homeostasis, (3) neutrophil EV‐mediated cytokine neutralization and anti‐inflammation, and (4) the maintenance of energy metabolism. The Combination of this nanoplatform with EVLP matches “disease‐specific” treatment plans. Furthermore, it uses a critical time during lung perfusion to protect cells via multiple mechanisms. With the availability of improved EVLP equipment in transplant centers, this method will not only help prevent early lung damage after surgery but also make more high‐risk donor lungs suitable for transplantation.

## 4 Limitation

While this study employed syngeneic rat transplantation to investigate protection against pulmonary IRI during Ltx, allogeneic transplantation models offer greater clinical relevance. The potential of nanoparticles to provide long‐term pulmonary protection and mitigate complications such as chronic lung allograft dysfunction (CLAD) following lung transplantation remains to be investigated using allogeneic models. Small animal EVLP is widely used due to its simplicity, convenience, and cost‐effectiveness, and it plays an important role in developing and testing lung protection strategies. However, small animal EVLP has limitations, such as differences in organ size, perfusion pressures not fully replicate clinical conditions. The large animal EVLP models warrant further development to explore lung injury repair that more closely mimics clinical scenarios during pulmonary transplantation.

## 5 Conclusion

This study demonstrated that SOD2‐Fer‐1@CVs, a hybrid nanoplatform integrating neutrophil‐derived EVs with ROS‐responsive liposomes, effectively mitigated IRI‐driven lung injury through multimodal mechanisms. By synergistically targeting oxidative stress, ferroptosis, and pro‐inflammatory cascades, the platform restores endothelial viability and enhances macrophage M2 polarization, eventually reducing lung injury. The integration of SOD2‐Fer‐1@CVs with our custom small‐animal EVLP system significantly improved alveolar‐capillary barrier function and suppressed lung injury, highlighting its potential for donor lung reconditioning. Innovatively combining a neutrophil‐mimetic target, ROS‐triggered drug release, and dual antioxidative/anti‐ferroptotic activity, this strategy represents a paradigm shift in “pathology‐tailored” nanomedicine. By addressing the multifactorial IRI pathology, SOD2‐Fer‐1@CVs‐enhanced EVLP has emerged as a transformative approach to expand donor lung utilization, reduce the incidence of PGD, and improve post‐transplant survival, offering a roadmap for next‐generation organ repair technologies.

## 6 Experimental Section

Please refer to the online Supporting Information for additional details on methodology.

### Cell Culture

HPMECs were obtained from Eallbio (cat.no., PC.00001). RAW264.7 cells were purchased from Eallbio. Human promyelocytic leukemia cells (HL60) were acquired from Procell (cat.no., CL‐0110). HL60 cells were transfected with an adenovirus encoding SOD2 to generate SOD2‐overexpressing HL60 cells (SOD2‐HL60). Cell culture dishes/plates, centrifuge tubes, and cryovials were obtained from NEST Biotechnology.‌ Puromycin dihydrochloride was obatained from Coolaber Science & Technology.

### Preparation of SOD2@Neu‐EVs, Fer‐1@DTP, and SOD2‐Fer‐1@CVs

SOD2‐overexpressing HL60 cells (SOD2‐HL60) were induced to differentiate into neutrophil‐like cells using 1.25% dimethyl sulfoxide for 4 days. The cell lysate was sonicated and sequentially filtered through polycarbonate membranes to collect SOD2@Neu‐EVs with a size range of 100–200 nm. Ferrostatin‐1 (Fer‐1, MCE, cat.no., HY‐100579), DSPE‐TK‐PEG2000 (RUIXIBIO, cat.no., R‐D526), cholesterol, and phosphatidylcholine (Solarbio, cat.no., IC0370) (mass ratio 1:1:1:5) were dissolved in chloroform, and a thin film was formed via rotary evaporation. The film was then hydrated with phosphate‐buffered saline (PBS) and homogenized via sonication for 20 min. The resulting suspension was filtered to obtain Fer‐1‐loaded nanoliposomes (Fer‐1@DTP). The two components (SOD2@Neu‐EVs and Fer‐1@DTP) were mixed in 1:1 weight ratio to obtain hybrid vesicles^[^ ^47^ ^]^ (SOD2‐Fer‐1@CVs).

### Characterization of Vesicles

TEM and DLS (Litesizer, Anton Paar) were used to determine the morphology, diameter, and zeta potential of the vesicles. The encapsulation efficiency and drug release profile of SOD2‐Fer‐1@CVs were assessed after stimulation with 50 µM H_2_O_2_ or without stimulation. The concentration of Fer‐1 was determined using a UV–vis spectrophotometer.

DiO‐labeled SOD2@Neu‐EVs and DiR@DTP were mixed and extruded through polycarbonate membranes to obtain DiO‐DiR@CVs. The fusion was assessed using confocal microscopy.SOD2‐Fer‐1@CVs were incubated with cytokines, IL‐6, IL‐1β, and TNF‐α, and cytokines in the supernatant were quantified using Enzyme‐Linked Immunosorbent Assay kits (ELISA) (Huabio).

### Cellular Uptake

DiO‐labeled SOD2‐Fer‐1@CVs (DiO‐labeled CVs) were prepared. HPMECs pretreated with TNF‐α were further treated with 1 µg mL^−1^ filipin (GlpBio, Cat.NO.GC18406), 30 U mL^−1^ chloroquine (GlpBio, cat.no., GC18406), 10 µg mL^−1^ chlorpromazine (GlpBio, cat.no., GC20060), or incubated at 4 °C for 1 h. After incubation with the DiO‐labeled CVs for 4 h, the DiO fluorescence in HPMECs was observed using confocal microscopy.

### Cell Proliferation, Apoptosis, and Mitochondrial Assays

For the cell experiments, the concentrations used were as follows: SOD2@Neu‐EVs (50 µg ml^−1^), Fer‐1@DTP (Fer‐1: 1 µM), and SOD2‐Fer‐1@CVs (50 µg ml^−1^; Fer‐1:1 µM). The effect of CVs on endothelial cell proliferation was evaluated using the cell counting Kit‐8 (CCK‐8) (Beyotime, cat.no., C0041) and 5‐bromo‐2′‐deoxyuridine (BrdU) (final concentration: 40 µM, Servicebio, cat.no., GC310002) assay. Apoptosis was detected using a TUNEL assay kit (Servicebio) according to the manufacturer's instructions. Mitochondrial morphology was observed and imaged using a transmission electron microscope (UC7, Leica). Cell apoptosis was assessed using an Annexin V/7‐AAD kit (Tonbo, cat.no., 35‐640‐KIT). Intracellular ROS levels were measured using the fluorescent probe DCFH‐DA (MeilunBio, cat.no., MA0219). Mitochondrial membrane potential was assessed using a JC‐1 staining kit (Beyotime, cat.no., C2003S).

### Macrophage Polarization Assay

RAW264.7 cells were polarized to M1 macrophages using LPS. Cells were fixed, permeabilized, and stained with an APC‐conjugated iNOS antibody (BioLegend, cat.no., 696 807) and phycoerythrin‐conjugated ARG‐1 antibodies (BioLegend, cat.no., 165 803) for 30 min at room temperature in the dark. Macrophage polarization was analyzed using flow cytometry.

### qRT‐PCR

Total RNA was extracted using an RNA extraction kit (Vazyme, cat.no., RC112). cDNA was synthesized and amplified using a PCR kit (Takara, cat.no., RR036A) with gene‐specific primers. Gene expression was quantified using a qPCR instrument (Bio‐Rad).

### Immunofluorescence Staining

For immunofluorescence staining of adherent cells (HPMECs and RAW264.7), the cells were fixed and blocked. Paraffin‐embedded tissue sections were prepared for lung tissue analysis. Primary antibodies against GPX4 (Servicebio, cat.no., GB124327), COX2 (Servicebio, cat.no., GB155672), CD86 (Servicebio, cat.no., GB13586), CD206 (Servicebio, cat.no., GB113497), CD31 (Servicebio, cat.no., GB11063), iNOS (Servicebio, cat.no., GB11119), and CD206 (Affinity, cat.no., DF4149) were incubated with the cells or tissue sections. The appropriate fluorescent secondary antibodies (Servicebio) were used. Fluorescence images were captured using a fluorescence microscope.

### ELISA

The levels of inflammatory cytokines (IL‐6, IL‐1β, and TNF‐α) were quantified using commercial ELISA kits (NeoBioscience, Cat. No. ERC003.96, ERC007.96, and ERC102a.96). MDA levels were measured using a commercial kit (Beyotime, Cat. No. S0131). SOD activity was determined using a WST‐8‐based assay kit (Beyotime, Cat. No. S0103).

### Western Blot Analysis

CVs, cell lysates, and lung tissue lysates were prepared using RIPA buffer supplemented with protease inhibitors. Protein samples were analyzed using a fully automated protein expression system (WESTM, Proteinsimple). Primary antibodies against SOD2 (Proteintech, Cat. No. 24127‐1‐AP), SCL7A11 (HUABIO, Cat. No. HA721868), ACSL4 (HUABIO, Cat. No. ET7111‐43), and β‐actin (Proteintech, Cat. No. 66009‐1‐Ig) were diluted 1:200 (1:2000 for β‐actin) and incubated with samples. Protein expression was quantified using Compass for SW software (Version 6.3.0).

### Rat Lung IRI Model

Male Sprague‐Dawley rats (350–400 g) were purchased from Guangdong Vital River Laboratory Animal Technology Co., Ltd. and housed in the Experimental Animal Center of Guangzhou Medical University. All experimental procedures were approved by the Experimental Animal Ethics Committee of the First Affiliated Hospital of Guangzhou Medical University (No. 20 250 044).

Rats were anesthetized with isoflurane and maintained with 3% pentobarbital sodium (50 mg kg^−1^, intraperitoneal injection). After tracheal intubation, the left pulmonary hilum was exposed, and the left pulmonary artery was clamped for 1 h to induce ischemia. Reperfusion was initiated by removing the clamp, and tissues were collected after 2 h.^[^ ^60^ ^]^ Sham‐operated rats underwent the same procedure without clamping. SOD2@Neu‐EVs (0.5 mg kg^−1^), Fer‐1@DTP (Fer‐1: 0.03 mg kg^−1^), and SOD2‐Fer‐1@CVs (0.5 mg kg^−1^; Fer‐1: 0.03 mg kg^−1^) were administered via the iliac artery before ischemia and after reperfusion to evaluate their protective effects against lung IRI.

### EVLP and Left Lung Transplantation—Donor Lung Procurement

Donor rats were anesthetized via isoflurane inhalation for induction, followed by an intraperitoneal injection of 1% amiodarone for maintenance. Tracheostomy was performed, and mechanical ventilation was initiated with 100% oxygen at a respiratory rate of 70 breaths min^−1^ and a tidal volume of 6 mL kg^−1^. The abdominal cavity was opened, and heparin (300 IU kg^−1^) was administered via the inferior vena cava to prevent blood clotting. The thoracic cavity was exposed via a midline sternotomy. The right atrium and inferior vena cava were incised to allow blood drainage, and the pulmonary artery was cannulated for cold perfusion. The lungs were perfused with a cold preservation solution at a hydrostatic pressure of 20 cm H_2_O.

### EVLP and Left Lung Transplantation—EVLP Procedure

A custom‐designed small‐animal EVLP system was used to simulate clinical EVLP. The system consisted of a perfusion circuit, ventilator, and temperature control unit. EVLP was performed as other described previously.^[^ ^61^, ^62^, ^63^ ^]^ The perfusion solution was oxygenated and maintained at 21 °C. It contained 5 g L^−1^ dextran 40, 40 g L^−1^ gelatin, 20 g L^−1^ rat serum albumin, 148 mmol L^−1^ Na⁺, 4 mmol L^−1^ K⁺, 103 mmol L^−1^ Cl^−^, 1 mmol L^−1^ Mg^2^⁺, 1 mmol L^−1^ Ca^2^⁺, 11 mmol L^−1^ glucose, and 24 mmol L^−1^ bicarbonate. Donor lungs were connected to the EVLP system, and perfusion was initiated at a low flow rate. Over the first 1.5 h, the perfusion flow rate was gradually increased to 20% of the estimated cardiac output. Mechanical ventilation was maintained with an inspiratory pressure of 15 cm H_2_O, a positive end‐expiratory pressure (PEEP) of 3 cm H_2_O, and a respiratory rate of 40 breaths per minute. The total duration of EVLP was 2 h.

### EVLP and Left Lung Transplantation—Left Lung Transplantation

The recipient rats were anesthetized using isoflurane inhalation and maintained with 1% amiodarone. Tracheostomy was performed, and mechanical ventilation was initiated as described for donor rats. The left thoracic cavity was exposed via a left lateral thoracotomy. The left pulmonary hilum of the recipient was clamped, and the left pulmonary artery, pulmonary vein, and bronchus were dissected. Custom‐made cuffs (16G for the pulmonary vein, 18G for the pulmonary artery, and 14G for the bronchus) were used to connect the donor lung to the recipient's vasculature and airway. The cuffs were secured with sutures, and the clamps were removed to restore blood flow and ventilation to the transplanted lung.

The Animals were divided into three groups: 1. LTx group: Left lung transplantation without EVLP, 2. EVLP+LTx group: Left lung transplantation following EVLP, 3. EVLP+SOD2‐Fer‐1@CVs+LTx group: Left lung transplantation following EVLP with the addition of SOD2‐Fer‐1@CVs (0.5 mg kg^−1^; Fer‐1: 0.03 mg kg^−1^) to the perfusion solution.

### In Vivo Biodistribution and Pharmacokinetics

DiR‐labeled or Dio‐labeled CVs were injected via the iliac artery immediately after left lung IRI. Fluorescence intensity was measured using an imaging system (Revvity). Plasma Dio‐EV concentration was quantified using a nanoflow cytometer (Apogee).

### Lung Injury Assay

Lung tissues were stained with H&E, and lung injury scores were calculated. The wet‐to‐dry weight ratio was calculated to assess pulmonary edema. Total cell counts in the BALF were determined using a cell counter. Oxygen concentration and partial pressure were analyzed using a blood gas analyzer.

### Safety Evaluation and Hemolysis Assay

Histopathological changes in the organs were assessed by the H&E method after injecting CVs. A fully automated biochemical analyzer (Chemary 800, Rayto) was used to measure alanine aminotransferase (ALT), aspartate aminotransferase (AST), urea, and creatinine levels. Red blood cells were collected and incubated with SOD2‐Fer‐1@CVs to calculate the hemolysis rate. Triton X‐100 and PBS were used as positive and negative controls, respectively.

### RNA Sequencing and Bioinformatic Analysis

Total RNA was extracted from the lung tissues of the IRI, IRI+SOD2‐Fer‐1@CVs, EVLP, and EVLP+SOD2‐Fer‐1@CVs groups. RNA quality was assessed using an Agilent 2100 Bioanalyzer, and libraries were prepared for sequencing on an Illumina platform. Raw data were processed, and differentially expressed genes were identified using DESeq2 (adjusted p < 0.05, |log2(fold change) | ≥ 0). Functional enrichment analysis was performed using the GSEA network.

### Statistical Analysis

Statistical analysis was performed using the Prism software package (PRISM 7.0). Data were presented as mean ± standard deviation. Comparisons between two groups were performed using the Student's t‐test, while multiple groups (more than two groups) were compared using one‐way analysis of variance (ANOVA) followed by Tukey's post‐hoc test. The sample size (n) for each experiment is described in the figure legends. Statistical significance was set at p < 0.05.

## Conflict of Interest

The authors declare no conflict of interest.

## Supporting information



Supporting Information

## Data Availability

The data that support the findings of this study are available from the corresponding author upon reasonable request.
