# Supplementary material for: Engineering Neutrophil Vesicles for Synergistic Protection against Ischemia/Reperfusion Injury after Lung Transplant
Source: Adv Sci (Weinh). 2025 Aug 14;12(42):e06127. doi: 10.1002/advs.202506127 (PMC12622495; doi:10.1002/advs.202506127)
Supplement: Supplementary file 1 — Supporting Information [file ADVS-12-e06127-s001.pdf]

Supporting Information

Engineering Neutrophil Vesicles for Synergistic Protection Against Ischemia/Reperfusion Injury After Lung Transplant

Hao-Xiang Yuan[1†], Yu-Yun Ye[2†], Pu Shen[3†], Jie Zhang[1†], Qian-Fang Meng[4], Ying Chen[1], Xin Xu[1], Xuan-Lin Zhang[1], Lang Rao[4]*, Zhi-Jin Fan[2]*, and Jian-Xing He[1,5]*

1. Department of Thoracic Surgery and Oncology, the First Affiliated Hospital of Guangzhou Medical University, State Key Laboratory of Respiratory Disease & National Clinical Research Center for Respiratory Disease, Guangzhou, 510120, China
2. Institute for Engineering Medicine, Kunming Medical University, Kunming, 650500, China.
3. Department of Anesthesiology, The First Affiliated Hospital, Sun Yat-sen University, Guangzhou, 510080, China
4. Institute of Chemical Biology, Shenzhen Bay Laboratory, Shenzhen, 518132, China
5. Southern Medical University, Guangzhou 510120, China

[†]These authors contributed equally to this work

*Corresponding author: Jian-Xing He, drjianxing.he@gmail.com; Lang Rao, lrao@szbl.ac.cn; Zhi-Jin Fan, fanzhijin@m.scnu.edu.cn.

Supplemental Methods and Materials

1. Cell Culture

HPMECs were obtained from Eallbio (Cat. No. PC.00001) and cultured in endothelial cell medium (ECM, Sciencell, Cat. No. 1001) supplemented with 10% fetal bovine serum (FBS) and 1% endothelial cell growth supplement (ECGS). RAW264.7 cells were purchased from Eallbio and maintained in Dulbecco's Modified Eagle Medium (DMEM) containing 10% FBS. Human promyelocytic leukemia cells (HL60) were acquired from Procell (Cat. No. CL-0110) and cultured in HL60-specific complete medium. To generate SOD2-overexpressing HL60 cells (SOD2-HL60) for subsequent preparation of SOD2@Neu-EVs, HL60 cells were transfected with adenovirus encoding SOD2. All cells were cultured at 37°C with 5% CO_2. To detect the uptake of SOD2-Fer-1@CVs in rat endothelial cells, the rat pulmonary microvascular endothelial cells (RPMECs, iCell Bioscience Inc, Shanghai, China) were purchased and cultured in ECM (10% FBS and 1% ECGS).

2. Preparation of SOD2@Neu-EVs, Fer-1@DTP, and SOD2-Fer-1@CVs

2.1 Preparation of SOD2@Neu-EVs

SOD2-overexpressing HL60 cells (SOD2-HL60) were induced to differentiate into neutrophil-like cells using 1.25% dimethyl sulfoxide (DMSO) for 4 days. The cells were collected, washed twice with phosphate-buffered saline (PBS), and resuspended in 1 mL PBS. Subsequently, 500 μL of hypotonic lysis buffer (1 mmol/L $NaHCO_3$, 0.2 mmol/L EDTA, and 1 mmol/L phenylmethylsulfonyl fluoride (PMSF)) was added, and the mixture was incubated overnight at 4°C. The lysate was sonicated and centrifuged at 3000 × g for 10 minutes at 4°C to remove debris. The supernatant was sequentially filtered through 800 nm, 400 nm, 200 nm, and 100 nm polycarbonate membranes to collect SOD2@Neu-EVs with a size range of 100–200 nm.

2.2 Preparation of Fer-1@DTP

Ferrostatin-1 (Fer-1, MCE, Cat.NO. HY-100579), DSPE-TK-PEG2000 (RUIXIBIO, Cat. NO. R-D526), cholesterol, and phosphatidylcholine (Solarbio, Cat.NO. IC0370) (mass ratio 1:1:1:5) were dissolved in chloroform, and a thin film was formed by rotary evaporation. The film was hydrated with PBS and homogenized by sonication for 20 minutes. Free Fer-1 was removed by centrifugation and filtration. The resulting suspension was sequentially filtered

through 400 nm, 200 nm, and 100 nm polycarbonate membranes to obtain Fer-1-loaded nanoliposomes (Fer-1@DTP) with reactive oxygen species (ROS)-responsive release properties.

2.3 Preparation of SOD2-Fer-1@CVs

The protein concentrations of SOD2@Neu-EVs and Fer-1@DTP were determined using a bicinchoninic acid (BCA) assay. The two components were mixed at a 1:1 weight ratio and extruded through 400 nm, 200 nm, and 100 nm polycarbonate membranes using a mini-extruder to obtain hybrid vesicles (SOD2-Fer-1@CVs) co-loaded with Fer-1 and SOD2 protein.

3. Characterization of Vesicles

3.1 Morphology and Size Analysis

The morphology and diameter of negatively stained SOD2-Fer-1@CVs were observed using transmission electron microscopy (TEM). The size distribution and zeta potential were measured using dynamic light scattering (DLS) (Litesizer, Anton Paar). To evaluate stability, the size changes of SOD2-Fer-1@CVs were monitored after storage at 4°C and 37°C for one week.

3.2 Encapsulation Efficiency and Drug Release

The encapsulation efficiency and drug release profile of SOD2-Fer-1@CVs were assessed using a centrifugal filtration device. The filtrate was collected at different time points (0, 0.5, 1, 2, 6, 12, 18, and 24 hours) after stimulation with 50 μM H_2O_2, and an equal volume of PBS was replenished at each time point. The concentration of Fer-1 in the filtrate was determined by measuring the absorption spectrum from 200 nm to 500 nm using a UV-visible spectrophotometer.

4. Cytokine Binding Assay

SOD2-Fer-1@CVs (100 μg/mL and 500 μg/mL) were incubated with inflammatory cytokines IL-6, IL-1β and TNF-α at 37°C for 1 hour. The mixture was centrifuged at 12,000 × g for 1 hour to remove CVs, and the levels of cytokines in the supernatant were quantified using ELISA kits (Huabio) according to the manufacturer's instructions.

5. Fusion Assessment

SOD2@Neu-EVs were labeled with DiO fluorescent dye to obtain DiO-Neu-EVs.

DiR@DTP was prepared by rotary evaporation and hydration using lecithin, cholesterol, DSPE-TK-PEG2000, and DiR dye. Free DiO and DiR dyes were removed by ultracentrifugation. The mixture was extruded through 400 nm, 200 nm, and 100 nm polycarbonate membranes to obtain DiO-DiR@CVs. The fusion was assessed by observing the fluorescence distribution of DiO and DiR using confocal microscopy.

6. Cellular Uptake

6.1 Fluorescence Labeling and Uptake Assay

SOD2-Fer-1@CVs were labeled with DiO fluorescent dye by incubation at room temperature for 30 minutes, and free DiO was removed by ultracentrifugation to obtain DiO-labeled SOD2-Fer-1@CVs. To investigate the effect of ROS levels on CVs uptake, HPMECs were stimulated with 10 ng/mL TNF-α (Novoprotein, Cat.NO. C008) to induce oxidative stress injury, followed by incubation with DiO-labeled CVs for 4 hours. After removing the supernatant, cells were washed with PBS, fixed with 4% paraformaldehyde (PFA), permeabilized with 0.1% Triton X-100, and stained with phalloidin and DAPI. The distribution and intensity of DiO fluorescence in cells were observed using confocal microscopy.

6.2 Mechanism of Cellular Uptake

To explore the mechanism of CVs uptake, HPMECs pretreated with TNF-α were further treated with 1 μg/mL filipin (GlpBio, Cat.NO.GC18406), 30 U/mL chloroquine (GlpBio, Cat.NO.GC18406), 10 μg/mL chlorpromazine (GlpBio, Cat.NO.GC20060), or incubated at 4°C for 1 hour. After incubation with DiO-labeled CVs for 4 hours, cells were washed, trypsinized, and were observed using confocal microscopy to determine the proportion of DiO-positive HPMECs.

7. Cell Proliferation Assays

7.1 CCK-8 Assay

To determine the impact of SOD2-Fer-1@CVs on HPMECs, we performed a CCK-8 assay to evaluate the effects of different concentrations of SOD2-Fer-1@CVs (0–150 μg/ml) on HPMECs with or without TNF-α stimulation. The Cell Counting Kit-8 (CCK-8) (Beyotime, Cat.NO.C0041) assay were performed to detect the endothelial cell proliferation.

The effect of Fer-1@DTP, SOD2@Neu-EVs and SOD2-Fer-1@CVs on endothelial cell proliferation was also evaluated by CCK-8 assay. In brief, HPMECs were seeded in 96-well

plates and treated with SOD2@Neu-EVs (50 μg/ml), Fer-1@DTP (Fer-1: 1 μM), and SOD2-Fer-1@CVs (50 μg/ml; Fer-1: 1 μM), and TNF-α. At 24, 48, and 72 hours, CCK-8 reagent was added, and cells were incubated at 37°C with 5% CO_2 for 2 hours. The absorbance at 450 nm was measured using a microplate reader.

7.2 BrdU Assay

Cell proliferation was further assessed using 5-bromo-2'-deoxyuridine (BrdU) incorporation. HPMECs were seeded in 6-well plates, and BrdU (final concentration: 40 μM, Servicebio, Cat.NO.GC310002) was added to the medium for 4 hours. Cells were fixed with 4% PFA, permeabilized with 0.1% Triton X-100, blocked with 1% bovine serum albumin (BSA), and incubated with an anti-BrdU antibody overnight at 4°C. After incubation with a fluorescent secondary antibody and DAPI staining, BrdU-positive cells were visualized using fluorescence microscopy.

8. TUNEL Assay

Apoptosis was detected using a TUNEL assay kit (Servicebio) according to the manufacturer’s instructions. Cells were washed with PBS, fixed with 4% PFA, and permeabilized with 0.1% Triton X-100 for 10 minutes at room temperature. The TUNEL reaction mixture was added, and cells were incubated at 37°C in the dark for 1 hour. After washing with PBS, nuclei were stained with DAPI, and TUNEL-positive cells were observed using fluorescence microscopy.

9. Transmission Electron Microscopy (TEM)

HPMECs were fixed with 2.5% glutaraldehyde and 1% osmium tetroxide, dehydrated in a graded series of ethanol and acetone, and embedded in resin. Ultrathin sections (70 nm) were prepared and stained with uranyl acetate and lead citrate. Mitochondrial morphology was observed and imaged using a transmission electron microscope (UC7, Leica).

10. Flow Cytometry Analysis

10.1 Apoptosis Detection

Cell apoptosis was assessed using an Annexin V/7-AAD kit (Tonbo, Cat. No. 35-640-KIT). Cells were resuspended at a density of 1×10^6 cells/mL, washed with PBS, and incubated with Annexin V binding buffer, Annexin V-FITC antibody, and 7-AAD for 30 minutes at room temperature in the dark. Apoptotic cells were analyzed by flow cytometry.

10.2 ROS Detection

Intracellular ROS levels were measured using the fluorescent probe DCFH-DA (MeilunBio, Cat. No. MA0219). Cells were washed with serum-free medium and incubated with 10 μM DCFH-DA at 37°C for 20 minutes in the dark. After washing, the fluorescence intensity of DCFH-DA was measured by flow cytometry.

10.3 Mitochondrial Membrane Potential Assay

Mitochondrial membrane potential was assessed using a JC-1 staining kit (Beyotime, Cat. No. C2003S). Cells were incubated with JC-1 working solution at 37°C for 20 minutes, washed, and analyzed by flow cytometry to detect JC-1 monomers (excitation: 490 nm, emission: 530 nm) and aggregates (excitation: 525 nm, emission: 590 nm).

10.4 Macrophage Polarization Assay

RAW264.7 cells were polarized to M1 macrophages using lipopolysaccharide (LPS). Cells were fixed, permeabilized, and stained with APC-conjugated iNOS antibody (Biolegend, Cat. No. 696807) and PE-conjugated ARG-1 antibody (Biolegend, Cat. No. 165803) for 30 minutes at room temperature in the dark. Macrophage polarization was analyzed by flow cytometry.

11. Quantitative Real-Time PCR (qRT-PCR)

Total RNA was extracted from HPMECs and RAW264.7 cells using an RNA extraction kit (Vazyme, Cat. No. RC112). cDNA was synthesized and amplified using a PCR kit (Takara, Cat. No. RR036A) with gene-specific primers. Gene expression was quantified using a qPCR instrument (Bio-Rad). Primer sequences are listed in the original text.

TNF-α (Mouse): F: CAGGCGGTGCCTATGTCTC, R: CGATCACCCCGAAGTTCAGTAG

IL-1β (Mouse): F: GAAATGCCACCTTTTGACAGTG, R: TGGATGCTCTCATCAGGACAG

IL-6 (Mouse): F: CTGCAAGAGACTTCCATCCAG, R: AGTGGTATAGACAGGTCTGTTGG

iNOS (Mouse): F: GTTCTCAGCCCAACAATACAAGA, R: GTGGACGGGTCGATGTCAC

ARG1 (Mouse): F: CTCCAAGCCAAAGTCCTTAGAG, R: GGAGCTGTCATTAGGGACATCA

GAPDH (Mouse): F: AGGTCGGTGTGAACGGATTTG, R: GGGGTCGTTGATGGCAACA

IL-6 (Human): F: ACTCACCTCTTCAGAACGAATTG, R:

CCATCTTTGGAAGGTTCAGGTTG

TNF-α (Human): F: CCTCTCTCTAATCAGCCCTCTG, R: GAGGACCTGGGAGTAGATGAG

IL-1β (Human): F: ATGATGGCTTATTACAGTGGCAA, R: GTCGGAGATTCGTAGCTGGA

SOD2 (Human): F: AACCCAAAGGGGAGTTGCTG, R: GAGCCTTGGACACCAACAGA

GAPDH (Human): F: GGAGCGAGATCCCTCCAAAAT, R: GGCTGTTGTCATACTTCTCATGG

12. Immunofluorescence Staining

For immunofluorescence staining of adherent cells (HPMECs and RAW264.7 cells), cells were fixed with 4% paraformaldehyde (PFA) and blocked with goat serum for 30 minutes. For lung tissue analysis, paraffin-embedded lung tissue sections were prepared, fixed, and permeabilized before incubation with primary antibodies. Primary antibodies, including GPX4 (Servicebio, Cat. No. GB124327), COX2 (Servicebio, Cat. No. GB155672), CD86 (Servicebio, Cat. No. GB13586), CD206 (Servicebio, Cat. No. GB113497), CD31 (Servicebio, Cat. No. GB11063), iNOS (Servicebio, Cat. No. GB11119), and CD206 (Affinity, Cat. No. DF4149), were diluted according to the manufacturer's instructions and incubated with cells or tissue sections overnight at 4°C. After washing, appropriate fluorescent secondary antibodies (Servicebio) were applied and incubated for 1 hour at room temperature. Nuclei were counterstained with DAPI, and slides were mounted with anti-fade mounting medium. Fluorescence images were captured using a fluorescence microscope.

13. Enzyme-Linked Immunosorbent Assay (ELISA)

Plasma and bronchoalveolar lavage fluid (BALF) were collected from experimental animals. Samples were centrifuged to remove debris, and supernatants were stored at -80°C until analysis. The levels of inflammatory cytokines (IL-6, IL-1β, and TNF-α) were quantified using commercial ELISA kits (NeoBioscience, Cat. No. ERC003.96, ERC007.96, and ERC102a.96) according to the manufacturer's instructions. Briefly, standards and diluted samples were added to 96-well plates and incubated for 90 minutes at room temperature in the dark. After washing, biotinylated antibody working solution was added and incubated for 60

minutes. Plates were washed again, and enzyme conjugate working solution was added for 37 minutes. Finally, substrate solution was added, and the reaction was stopped after 15 minutes. Absorbance at 450 nm was measured using a microplate reader.

14. Malondialdehyde (MDA) and Superoxide Dismutase (SOD) Assays

Cell pellets and lung tissues were homogenized in lysis buffer and centrifuged at 13,000 × g for 15 minutes at 4°C. Supernatants were collected, and protein concentrations were determined using a BCA protein assay kit.

MDA levels were measured using a commercial kit (Beyotime, Cat. No. S0131). Briefly, 0.1 mL of sample or standard was mixed with 0.2 mL of MDA detection working solution and heated in a boiling water bath for 15 minutes. After cooling, samples were centrifuged at 1,000 × g for 10 minutes, and the absorbance of the supernatant was measured at 532 nm.

SOD activity was determined using a WST-8-based assay kit (Beyotime, Cat. No. S0103). Samples were mixed with SOD detection buffer, WST-8/enzyme working solution, and reaction initiation solution, followed by incubation at 37°C for 30 minutes. Absorbance at 450 nm was measured, and SOD activity was calculated based on the standard curve.

15. Western Blot Analysis

CVs, cell lysates, and lung tissue lysates were prepared using RIPA buffer supplemented with protease inhibitors. Protein concentrations were determined using a BCA assay. Protein samples (1 μg/μL) were loaded into designated wells of a detection plate (Cat. No. SM-W002) and analyzed using a fully automated protein expression system (WESTM, Proteinsimple). Primary antibodies against SOD2 (Proteintech, Cat. No. 24127-1-AP), CD11b (Servicebio, Cat.NO. GB15058), CXCR2(HUABIO, Cat.NO. ER1906-87), CXCR4(HUABIO, Cat.NO. HA722304), SCL7A11 (HUABIO, Cat. No. HA721868), ACSL4 (HUABIO, Cat. No. ET7111-43), and β-actin (Proteintech, Cat. No. 66009-1-Ig) were diluted 1:200 (1:2000 for β-actin) and incubated with samples. Secondary antibodies, streptavidin-HRP, and chemiluminescent substrate were applied according to the manufacturer's instructions. Protein expression was quantified using Compass for SW software (Version 6.3.0).

16. In Vivo Biodistribution and Pharmacokinetics

DiR-labeled CVs were injected via the iliac artery immediately after left lung IRI. At 0, 1- and 2 hours post-reperfusion, major organs (heart, liver, spleen, lungs, and kidneys) were

collected, and fluorescence intensity was measured using an imaging system.

Dio-labeled CVs were injected, and blood samples were collected at 0, 0.5, 1, 1.5, and 2 hours. Plasma was separated by gradient centrifugation, and Dio-EV concentrations were quantified using nanoflow cytometry (Apogee).

17. Histopathology and Lung Injury Scoring

Lung tissues were fixed in 4% PFA, paraffin-embedded, and sectioned at 5 μm thickness. Sections were stained with hematoxylin and eosin (H&E). Two blinded pathologists evaluated H&E-stained sections based on neutrophil infiltration, hyaline membrane formation, protein debris, and alveolar septal thickening. A score of 0–1 was assigned to each parameter, and six random fields per lung were analyzed.

18. Pulmonary Vascular Leakage Assessment

Lung tissues were weighed immediately after collection (wet weight) and after drying in a 60°C oven for 48 hours (dry weight). The wet-to-dry weight ratio was calculated to assess pulmonary edema. BALF was collected by instilling 2 mL of 0.9% NaCl into the trachea and aspirating three times. Total cell counts in BALF were determined using a cell counter.

19. Blood Gas Analysis

Arterial blood was collected from the left ventricle and analyzed using a blood gas analyzer to measure oxygen concentration and partial pressure.

20. In Vivo Safety Evaluation

Tissues (heart, liver, spleen, lungs and kidneys) were fixed, paraffin-embedded, and stained with H&E. Histopathological changes, including inflammatory cell infiltration and tissue damage, were evaluated under a light microscope.

Blood samples were analyzed using a fully automated biochemical analyzer (Chemary 800, Rayto) to measure alanine aminotransferase (ALT), aspartate aminotransferase (AST), urea, and creatinine levels, assessing liver and kidney function.

21. Hemolysis Assay

Fresh rat blood was collected and centrifuged to isolate red blood cells (RBCs). RBCs were resuspended in PBS and incubated with SOD2-Fer-1@CVs at 37°C for 1 hour. After centrifugation, the absorbance of the supernatant at 540 nm was measured to calculate the hemolysis rate. Triton X-100 and PBS were used as positive and negative controls, respectively.

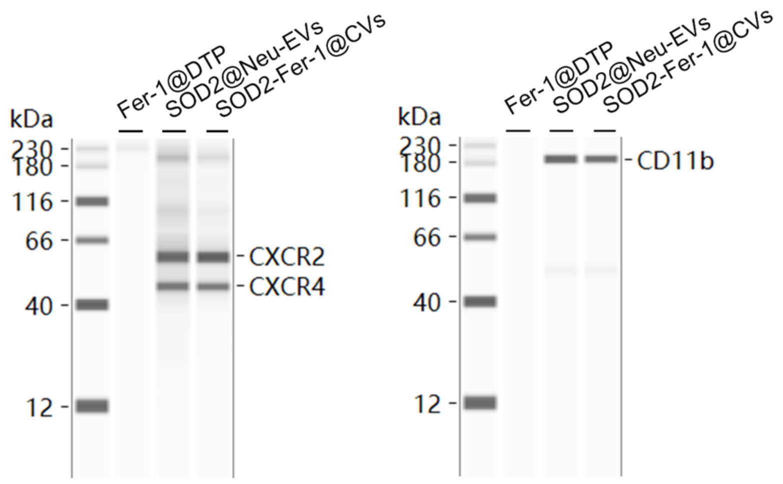

Supplemental figure 1. Identification of Neutrophil Markers

The Western blot was performed to detect the levels of neutrophil-related markers, such as CD11b and chemokine receptors (CXCR2 and CXCR4), in Fer-1@DTP, SOD2@Neu-EVs, and SOD2-Fer-1@CVs.

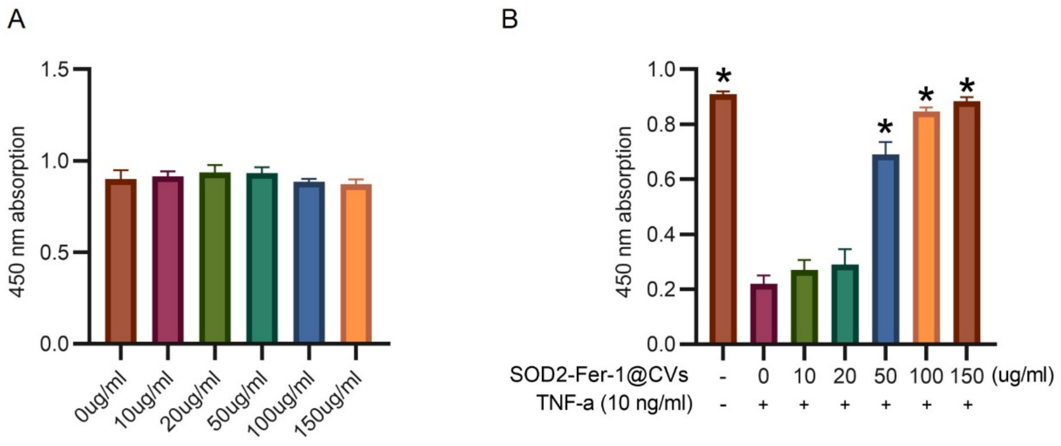

Supplemental figure 2. Effects of SOD2-Fer-1@CVs on the Proliferative Capacity of HPMECs

A) To determine the impact of SOD2-Fer-1@CVs on HPMECs, we performed a CCK-8 assay to evaluate the effects of different concentrations of SOD2-Fer-1@CVs (0–150 μg/ml) on HPMECs. The absorbance at 450 nm was measured after 3 days. B) To assess the protective effect of SOD2-Fer-1@CVs against TNF-α-induced impairment of HPMEC proliferation, HPMECs were pretreated with various concentrations (0–150 μg/ml) of SOD2-Fer-1@CVs, followed by TNF-α stimulation. The absorbance change was evaluated using the CCK-8 assay three days later. * vs. EVLP, $P < 0.05$, $n = 5$.

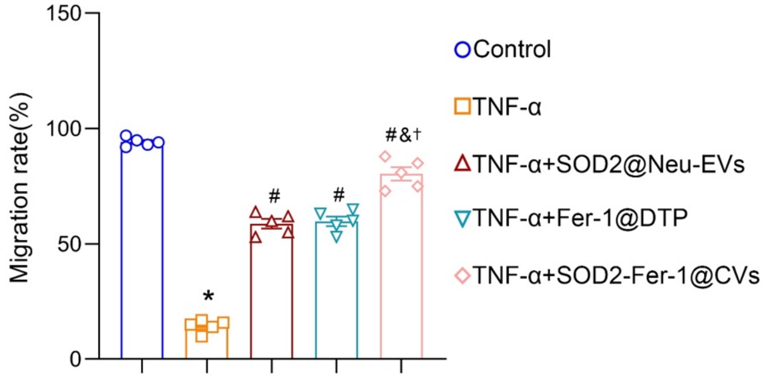

Supplemental figure 3. SOD2-Fer-1@CVs promote Endothelial migration

Scratch wound assay demonstrating migratory capacity of TNF-α-stimulated HPMECs pretreated with SOD2@Neu-EVs (50 μg/ml), Fer-1@DTP (Fer-1: 1 μM), or SOD2-Fer-1@CVs (50 μg/ml; Fer-1: 1 μM (* vs. Control; # vs. TNF-α; & vs. TNF-α+SOD2@Neu-EVs; † vs. TNF-α +Fer-1@DTP, $P < 0.05$, $n = 5$).

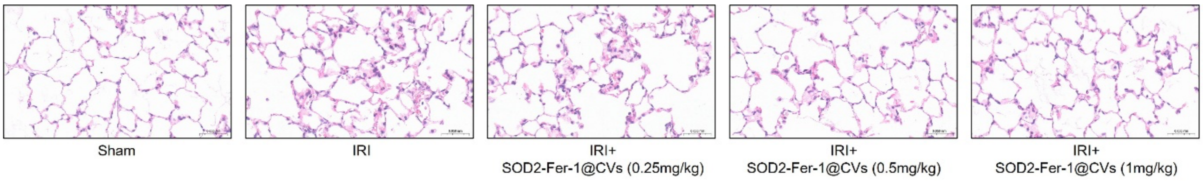

Supplemental figure 4. Protective Effects of Different Concentrations of SOD2-Fer-1@CVs on Lung IRI

To determine the optimal concentration of SOD2-Fer-1@CVs for alleviating lung IRI injury, we treated IRI model mice with SOD2-Fer-1@CVs at doses of 0.25, 0.5, and 1 mg/kg. Lung tissues were then harvested and subjected to H&E staining (scale bar: 50 μm).

Rat PMECs

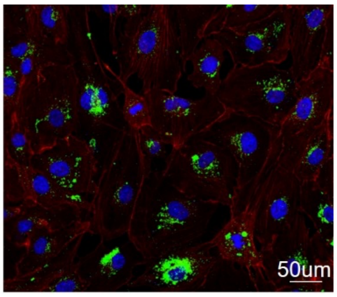

Supplemental figure 5. Uptake of SOD2-Fer-1@CVs by Rat Pulmonary Microvascular Endothelial Cells

Dio fluorescently labeled SOD2-Fer-1@CVs were added to rat pulmonary microvascular endothelial cells, and the uptake of CVs by the endothelial cells was observed after 3 hours. The cytoskeleton was stained with phalloidin (red), and nuclei were stained with DAPI (blue). Scale bar: 50 μm.

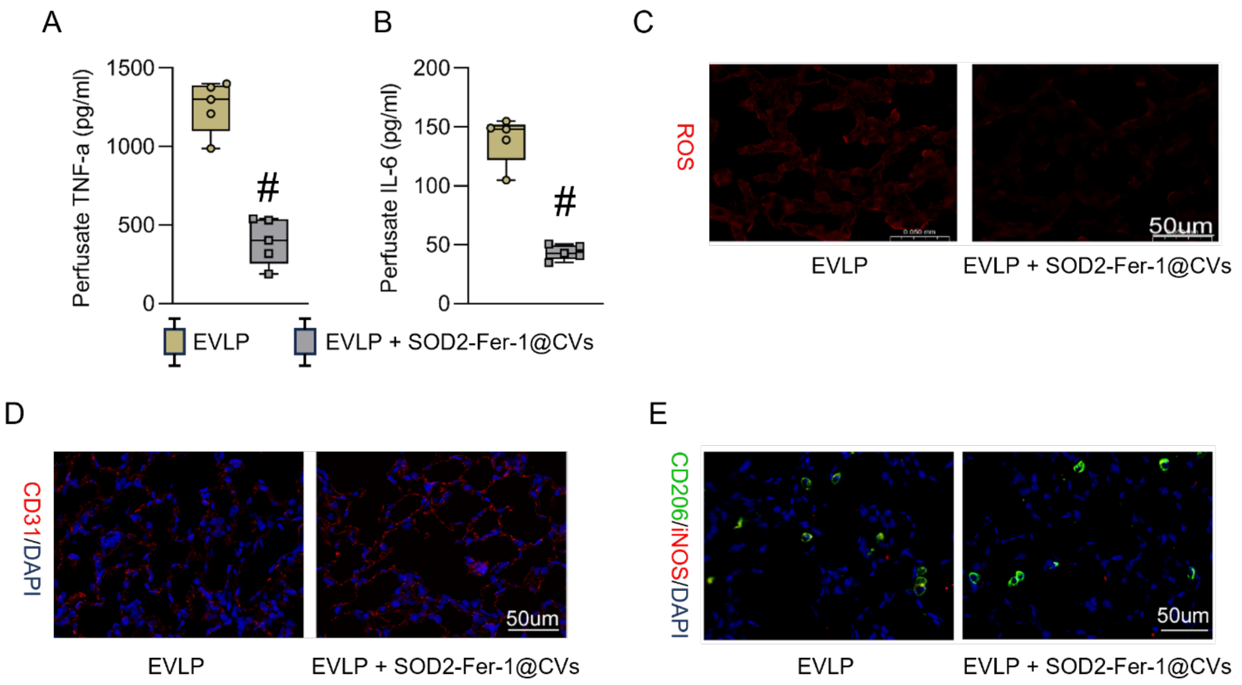

Supplemental figure 6. SOD2-Fer-1@CVs attenuate lung injury during Ex Vivo Lung Perfusion (EVLP)

The lungs were perfused in EVLP procedure with/without SOD2-Fer-1@CVs (0.5mg/kg; Fer-1: 0.03 mg/kg) before LTx. A-B) Cytokines levels of TNF-a and IL-6 in perfusate. C) pulmonary ROS quantification (red). D) $CD31^+$ endothelial integrity (red). E) Dual-polarization mapping of alveolar macrophages ($iNOS^+$ M1: red; $CD206^+$ M2: green; DAPI: blue. Scale bars:50um. # vs. EVLP, $P < 0.05$, $n = 5$).

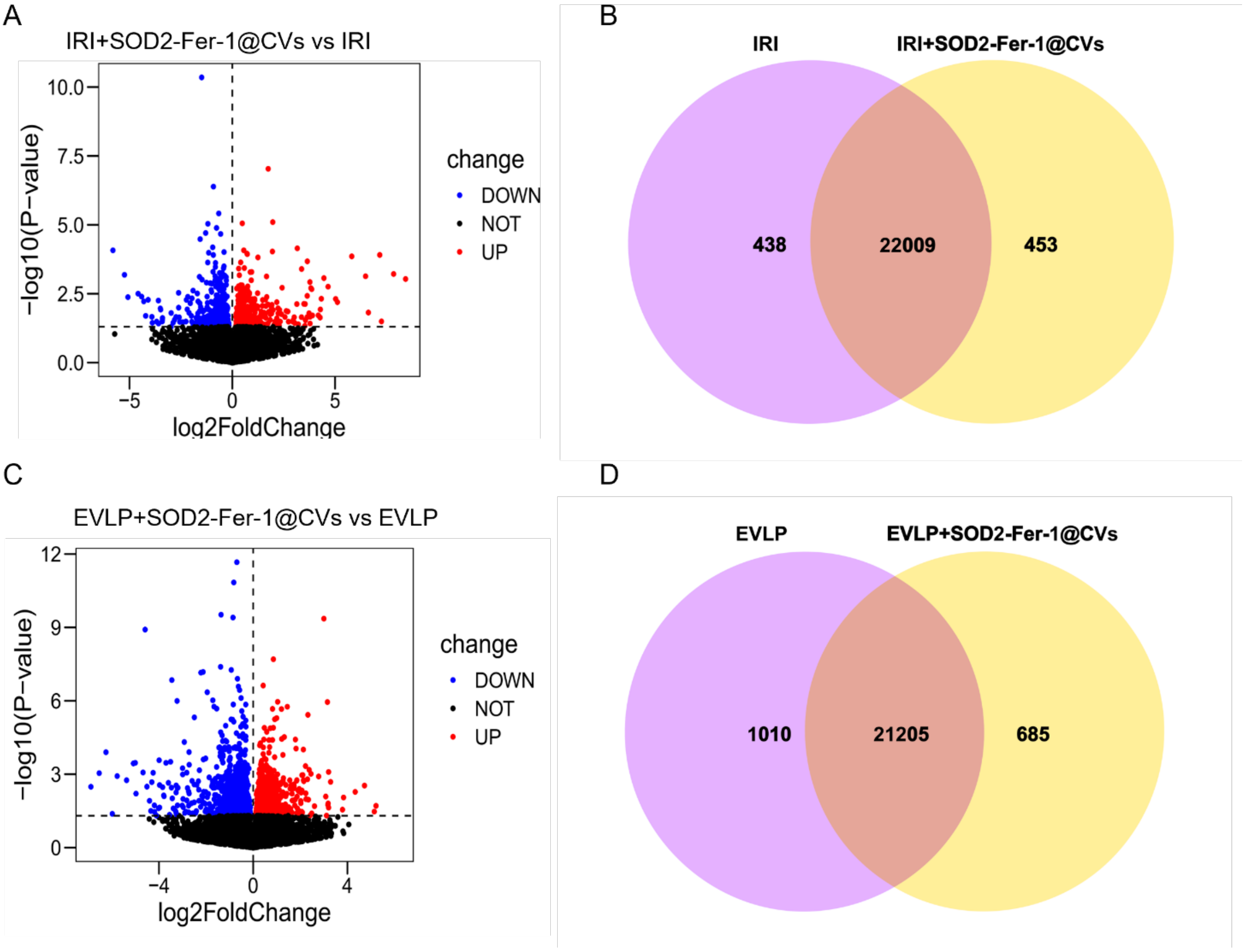

Supplemental figure 7. Transcriptional profiling analysis

A-B) Transcriptional profiling of SOD2-Fer-1@CVs (0.5mg/kg; Fer-1: 0.03 mg/kg) in ischemia-reperfusion injury (IRI) models by Volcano plot and Venn Diagram. C-D) Differential expressed gene of lung tissues in EVLP model with or without SOD2-Fer-1@CVs.

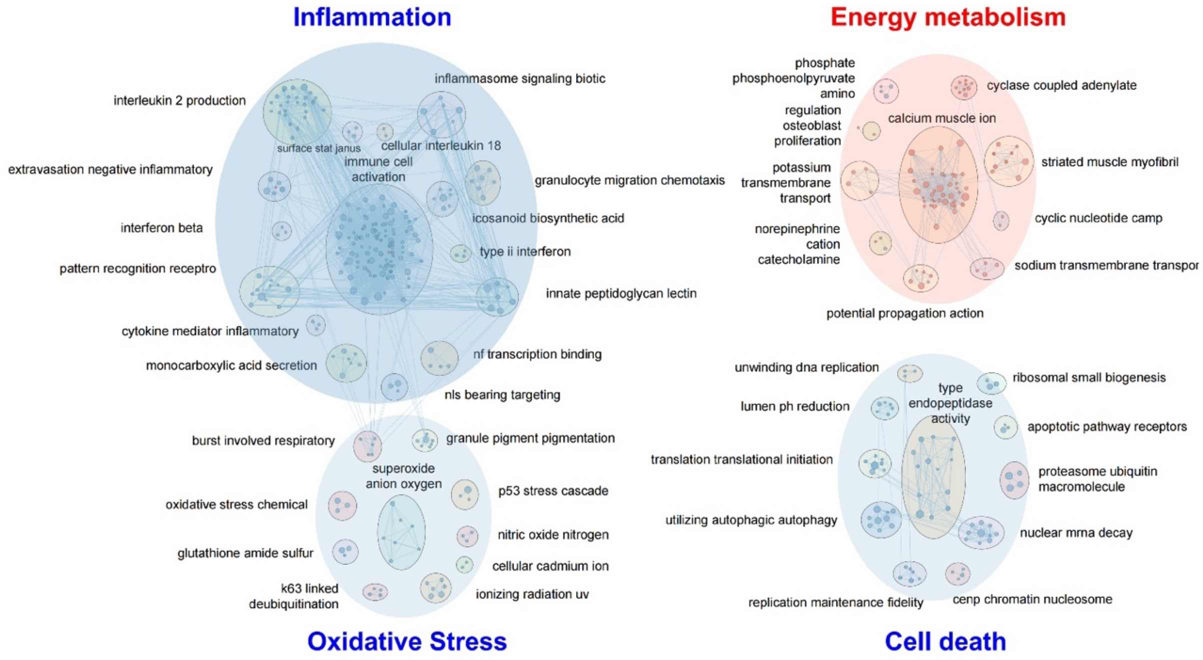

Supplemental figure 8. Transcriptomic Insights into SOD2-Fer-1@CVs-Mediated Pulmonary Repair During IRI procedure

Transcriptional profiling of SOD2-Fer-1@CVs (0.5mg/kg; Fer-1: 0.03mg/kg) in ischemia-reperfusion injury (IRI) models were detected to calculate the differential express genes. Gene Set Enrichment Analysis (GSEA) of differential express genes and network pharmacology to systematically elucidate the underlying mechanisms and molecular interaction networks associated with key biological processes such as inflammation, energy metabolism, oxidative stress, and regulated cell death (red: upregulated pathways; blue: downregulated pathways).

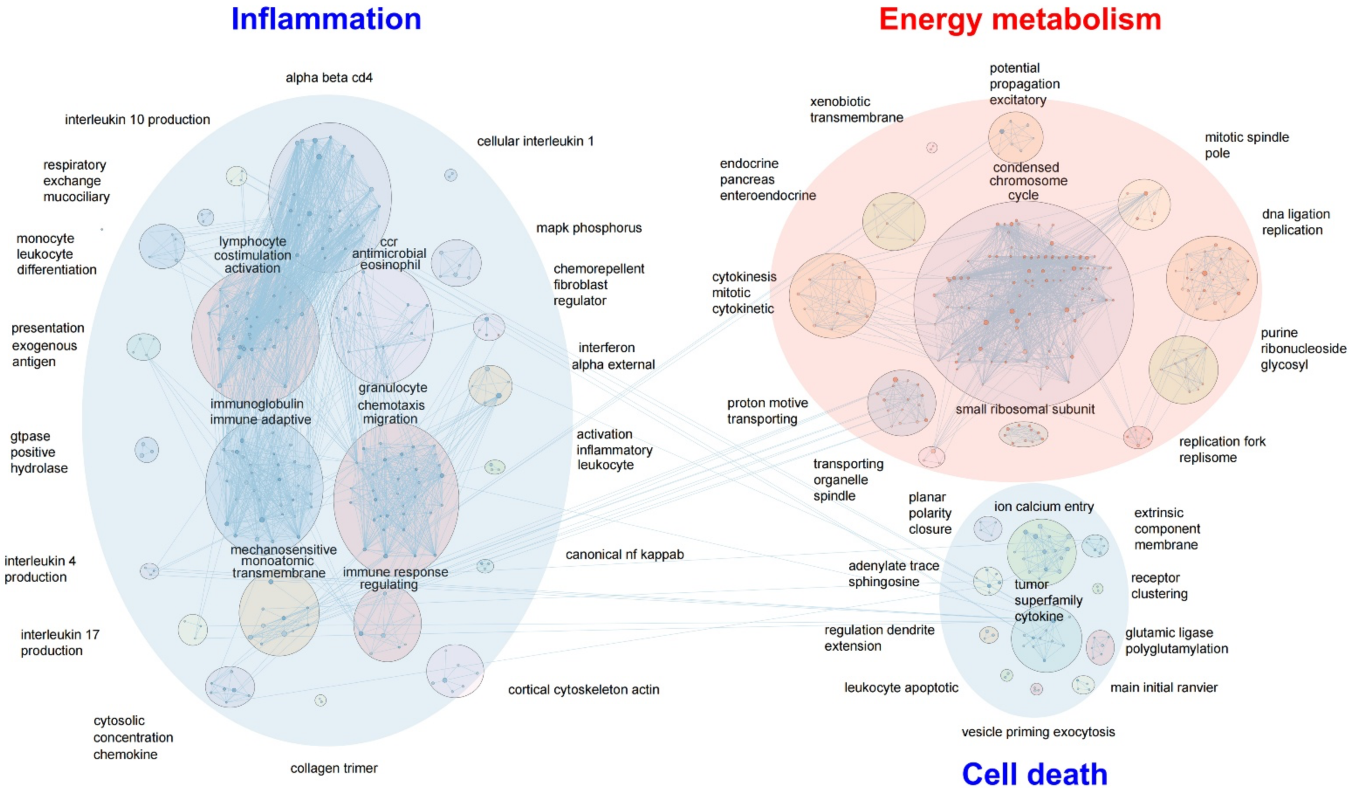

Supplemental figure 9. Transcriptomic Insights into SOD2-Fer-1@CVs-Mediated Pulmonary Repair During EVLP procedure

Transcriptional profiling of SOD2-Fer-1@CVs (0.5mg/kg; Fer-1: 0.03mg/kg) in EVLP models were detected to calculate the differential express genes. The GSEA network provides a systematic elucidation of the underlying mechanisms and molecular interaction networks involved in key biological processes, including inflammation, energy metabolism, and regulated cell death (with upregulated pathways shown in red and downregulated pathways in blue).

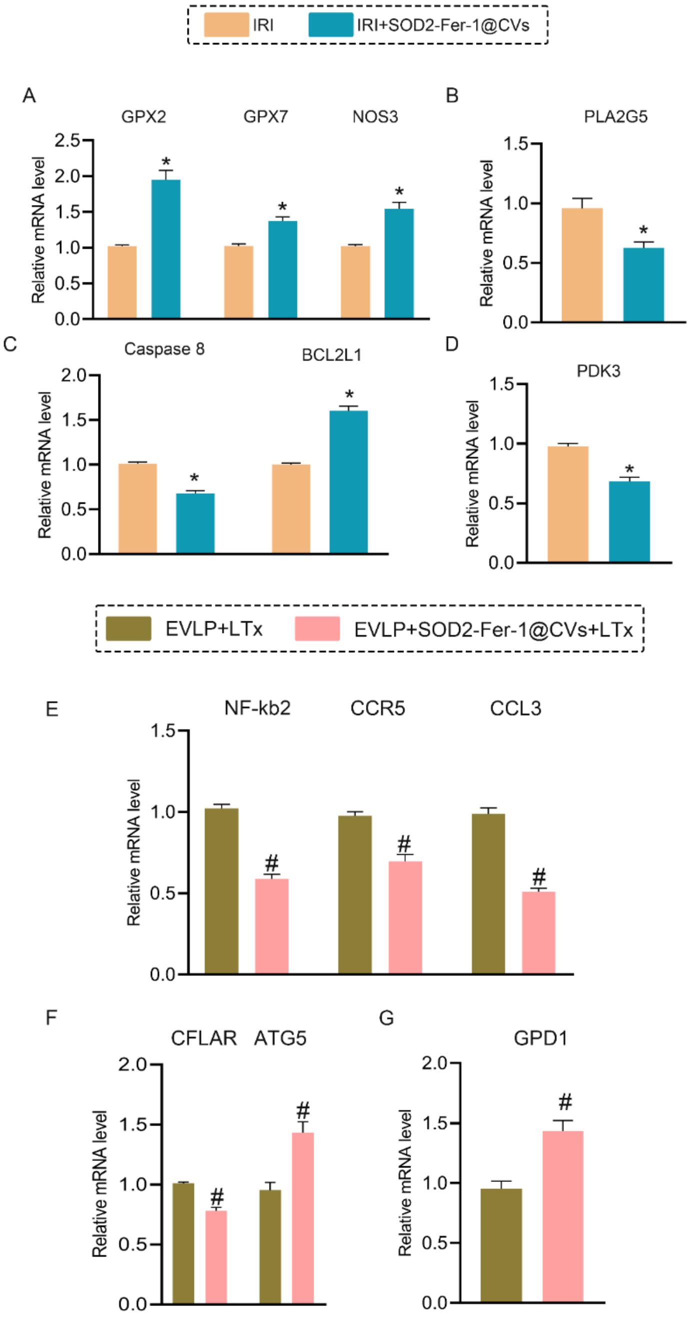

Supplemental figure 10. Validation of Sequencing Results

Based on the Gene Set Enrichment Analysis (GSEA) of lung tissue transcriptome sequencing following SOD2-Fer-1@CVs (0.5mg/kg; Fer-1: 0.03 mg/kg) treatment in IRI and EVLP+LTx models, we performed qRT-PCR to validate key genes involved in core processes such as inflammation, oxidative stress, energy metabolism, and cell death. A-D) mRNA levels of glutathione peroxidase 2 (GPX2), glutathione peroxidase 7 (GPX7), nitric oxide synthase 3

(NOS3), phospholipase A2 group V (PLA2G5), caspase 8, BCL2-like 1 (BCL2L1), and pyruvate dehydrogenase kinase isozyme 3 (PDK3) in IRI and IRI + SOD2-Fer-1@CVs groups. E-F) mRNA levels of nuclear factor kappa-light-chain-enhancer of activated B cells 2 (NF-κB2), C-C chemokine receptor type 5 (CCR5), C-C motif chemokine ligand 3 (CCL3), caspase 8 and FADD-like apoptosis regulator (CFLAR), autophagy related 5 (ATG5), and glycerol-3-phosphate dehydrogenase 1 (GPD1) in the EVLP + LTx model with or without SOD2-Fer-1@CVs (0.5mg/kg; Fer-1: 0.03 mg/kg) treatment. (* vs. IRI, # vs. EVLP+LTx, N=3, $P < 0.05$).

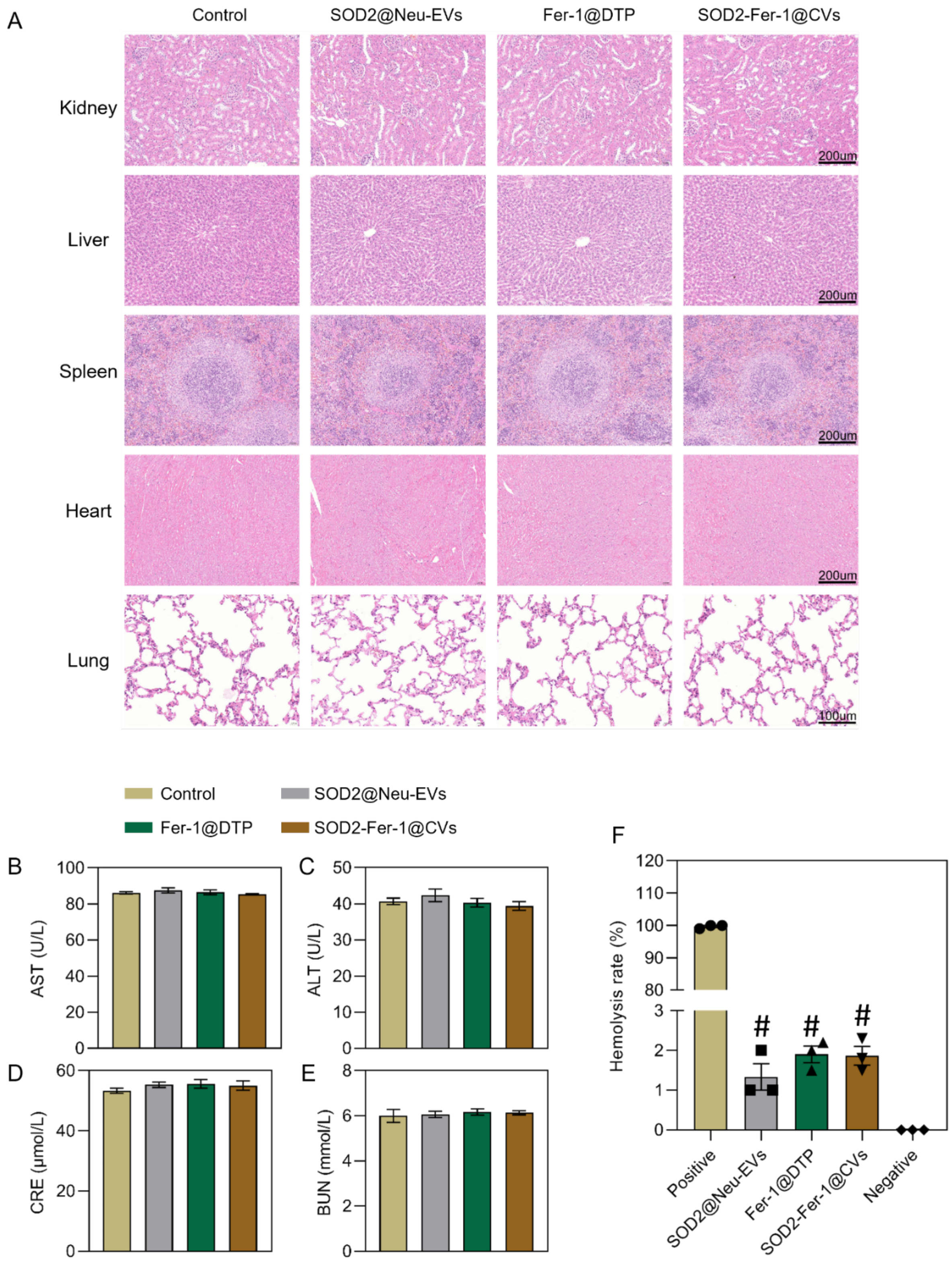

Supplemental figure 11. Biosafety Evaluation

A) To evaluate the in vivo biosafety of SOD2-Fer-1@CVs, H&E staining was performed on heart, liver, spleen, lung, and kidney tissues after SOD2-Fer-1@CVs (0.5mg/kg; Fer-1: 0.03 mg/kg) were administered via iliac artery injection in rats. B) Blood samples were collected

and analyzed using a biochemical analyzer to measure ALT, AST, BUN, and CRE levels. C) Hemolysis rate was assessed after incubating rat erythrocytes with SOD2-Fer-1@CVs for 3 hours. Triton X-100 and saline were used as positive and negative controls, respectively (# vs. positive control, $P < 0.05$, N=3).
